# The bidirectional association between obstructive sleep apnea and diabetic kidney disease: systematic review and meta-analysis

**DOI:** 10.3389/fendo.2025.1715997

**Published:** 2025-11-24

**Authors:** Jieyu Zhang, Yuandong Li, Bo Dai, Zheng Nan

**Affiliations:** 1 Changchun University of Chinese Medicine, Changchun, China; 2 The Affiliated Hospital of Changchun University of Chinese Medicine, Changchun, China

**Keywords:** obstructive sleep apnea, diabetic kidney disease, meta-analysis, systematic review, apnea-hypopnea index, estimated glomerular filtration rate, blood oxygen saturation

## Abstract

**Objective:**

This study aims to comprehensively explore the bidirectional association between obstructive sleep apnea (OSA) and diabetic kidney disease (DKD) through a systematic review and meta-analysis.

**Method:**

Systematically search for relevant literature on the association between OSA and DKD published from database inception to September 2025. Searches were performed in the Cochrane Library, PubMed, Embase, and Web of Science. Study quality was assessed with the Newcastle-Ottawa Scale (NOS). Meta-analysis, sensitivity analysis, and publication bias assessment were conducted using Review Manager 5.4, while R software was employed to calculate prediction intervals. The certainty of evidence was evaluated using the GRADE (Grading of Recommendations Assessment, Development, and Evaluation) framework.

**Result:**

A total of 14 articles involving 5,316 subjects were included. Patients with OSA exhibited a 1.92-fold increased risk of DKD compared with the control group (OR = 1.92, 95% CI: 1.59, 2.32, P < 0.0001). Subgroup analysis by OSA severity indicated a higher risk of DKD in patients with severe OSA (OR = 2.29) than in those with mild to moderate disease (OR = 1.52). Additionally, OSA patients showed significantly lower estimated Glomerular Filtration Rate (eGFR) levels (MD = -8.61, 95% CI: -12.92, -4.30) relative to non-OSA controls. In the reverse analysis, while the prevalence of OSA did not differ significantly between DKD and non-DKD groups (OR = 1.56, 95% CI: 0.71 - 3.43, P = 0.27), patients with DKD had significantly higher apnea-hypopnea index (AHI) (MD = 6.48, 95% CI: 1.74 - 11.22, P = 0.007) and lower average blood oxygen saturation (M-SaO2) (MD = -0.59, 95% CI: -0.82 to -0.36, P < 0.00001). No significant differences were observed in the lowest blood oxygen saturation (L-SaO_2_) between DKD and non-DKD groups across all subgroup analyses (all P > 0.05).

**Conclusion:**

A significant bidirectional association exists between OSA and DKD, suggesting a mutual exacerbation of risks between the two conditions. These findings highlight the clinical importance of enhanced OSA screening in diabetic populations and regular renal function monitoring in OSA patients.

## 1 Introduction

Obstructive sleep apnea (OSA) is a prevalent chronic sleep disorder characterized by recurrent upper airway collapse during sleep, which leads to intermittent hypoxemia (IH), hypercapnia, and sleep fragmentation (1). Common clinical manifestations encompass nocturnal snoring, frequent awakenings, impaired sleep quality, and excessive daytime sleepiness (2). Epidemiological data indicate a rapidly growing global burden, with current estimates suggesting approximately one billion affected adults. Recent population-based studies report prevalence rates reaching 26% (3, 4), particularly elevated among high-risk groups, including those with obesity or hypertension (5). Beyond its sleep-related manifestations, OSA is recognized as a multisystem disorder with clinical implications extending far beyond sleep medicine. Substantial evidence establishes its association with various comorbidities, particularly cardiovascular diseases, metabolic disorders, and neurocognitive impairment (2, 6).

Diabetic kidney disease (DKD) constitutes a primary microvascular complication of diabetes, affecting an estimated 30% to 40% of patients. This condition significantly increases cardiovascular mortality and represents the leading cause of end-stage renal disease (ESRD) (7). Pathologically, DKD manifests as progressive decline in glomerular filtration rate and elevated urinary albumin excretion (8), adversely affecting both quality of life and long-term patient survival.

The association between OSA and diabetes and its complications has garnered significant attention. Notably, the prevalence of OSA in individuals with diabetes exceeds 50%—a figure substantially higher than that observed in the general population (9). The condition directly disrupts glucose homeostasis through intermittent hypoxia and sleep fragmentation, which promote sympathetic overactivity, worsen insulin resistance, stimulate hepatic glucose production, and raise blood pressure (10). These effects are compounded by OSA-induced oxidative stress, triggering release of inflammatory cytokines such as tumor necrosis factor-α(TNF-α) and interleukin-6(IL-6), enhancing advanced glycation end products (AGEs) formation, and activating the renin–angiotensin–aldosterone system (RAAS) (11, 12). Collectively, these mechanisms induce endothelial dysfunction and microvascular injury (13). Critically, these pathways closely mirror those driving glomerular damage and renal interstitial fibrosis in DKD, suggesting OSA may contribute to DKD pathogenesis through both direct and indirect pathways.

While direct evidence supporting the bidirectional association between OSA and DKD remains limited, accumulating mechanistic and clinical investigations have progressively substantiated this hypothesis. Mechanistically, OSA may exacerbate renal injury through IH-mediated oxidative stress and sympathetic overactivation (14, 15). Animal studies have further demonstrated that chronic IH accelerates renal cell apoptosis and fibrosis in diabetic mice (16). Conversely, the potential impact of DKD on OSA warrants equal attention, as DKD-related fluid retention and upper airway edema may aggravate nocturnal airway obstruction, thereby establishing a pathological positive feedback loop (17, 18). Clinically, evidence from multiple observational studies has consistently linked the severity of OSA to key parameters of renal function: in obese diabetic patients, the apnea-hypopnea index (AHI) shows an inverse relationship with eGFR, with longer nocturnal hypoxia duration associated with more pronounced eGFR decline (19). Additionally, AHI has been identified as an independent predictor of both urinary albumin-to-creatinine ratio (UACR) and eGFR (20), with two cross-sectional studies confirming independent associations between OSA parameters and DKD or microalbuminuria (19, 21). Nevertheless, contradictory evidence exists. A small-scale study (n=52) failed to demonstrate a significant association between OSA and microalbuminuria (22), highlighting the need for further standardized research to elucidate this potential bidirectional relationship.

To address the considerable heterogeneity across existing studies in terms of sample characteristics, assessment methods, and interpretation of findings, we conducted a comprehensive meta-analysis to systematically evaluate the clinical association between OSA and DKD. We specifically investigated the impact of varying OSA severity levels on DKD risk, assessed alterations in OSA-related parameters in DKD patients, and elucidated the relationship between OSA and renal function indicators. This study provides crucial evidence to guide early clinical identification and targeted interventions in this high-risk population.

## 2 Materials and methods

### 2.1 Search strategy

This systematic review was conducted following the Preferred Reporting Items for Systematic Reviews and Meta-Analyses (PRISMA) guidelines (23) and the Meta-analysis of Observational Studies in Epidemiology (MOOSE) checklist (24) (**Supplementary Table 1**). Two independent researchers conducted a comprehensive search of electronic databases PubMed, Web of Science, Embase, and Cochrane Library from database inception to September 2025. The search strategy employed a combination of subject headings and free-text terms related to OSA “obstructive sleep apnea” or “sleep apnea syndrome” or “sleep-disordered breathing” or “OSA” or “OSAS” or “SAS” or “SDB”) and DKD (“diabetic kidney disease” or “Diabetic nephropathies” or “Nephropathies, Diabetic” or “Kidney diseases, Diabetic” or “Diabetic nephropathy”) (the full search strategy is provided in **Supplementary Table 2**). We also checked the published reviews and the reference lists of the included studies to determine potential publications that might meet the inclusion criteria. The study protocol is registered in the Prospective Register of Systematic Reviews (Prospero CRD420251157002).

### 2.2 Inclusion and exclusion criteria

Inclusion Criteria (1): Study Type: Observational studies, including cross-sectional studies, case-control studies, or cohort studies (both prospective and retrospective); (2) Study Subjects: Adult patients with type 2 diabetes (age≥18 years), regardless of gender; (3) Exposure and Outcome: If OSA is the exposure factor and DKD is the outcome indicator, OSA should be diagnosed through polysomnography (PSG) or portable sleep monitoring (ApneaLink) (25, 26); the diagnosis of DKD should be based on recognized clinical standards, including eGFR < 60 mL/min/1.73m², UACR ≥ 30mg/g, or medical records. If DKD is the exposure factor and OSA is the outcome indicator, the diagnostic criteria for DKD and OSA are the same as above.

Exclusion Criteria: (1) Republished and included studies; (2) Reviews, conference abstracts, editorials, comments, letters, case reports, case series studies that cannot be obtained in full text; (3) Non-human studies such as animal experiments, *in vitro* cell experiments, etc.; (4) Studies with missing data or unable to obtain valid data; (5) Studies with other primary diseases that may interfere with OSA and DKD outcomes, such as central sleep apnea, end-stage renal disease (ESRD), severe cardiopulmonary failure, malignant tumors, etc.; (6) Studies with subjects currently receiving or having received OSA-specific treatments such as continuous airway positive pressure ventilation, or using drugs that may significantly affect sleep structure or renal function; (7) Studies where OSA diagnosis is only based on the Epworth Sleepiness Scale or self-reported snoring symptoms assessment; (8) Studies not published in English.

### 2.3 Literature screening and data extraction

Two researchers (J.Z. and Y.L.) independently performed literature screening and data extraction. First, search results from all databases were merged using EndNote, and duplicates were removed. The researchers then screened titles and abstracts to exclude obviously irrelevant studies. For potentially eligible studies, full texts were retrieved and reviewed against predefined inclusion and exclusion criteria. Disagreements were resolved through discussion, with a third researcher (D.B.) arbitrating unresolved issues.

For the included studies, two researchers independently extracted data using a predesigned data extraction form. All extracted data were cross-verified and included the following information: first author, publication year, country, study design, patient characteristics (including sample size, age, sex ratio, BMI, and confounding factors), definitions of OSA and DKD, prevalence of OSA and DKD, nocturnal oxygen parameters, and renal function indicators.

### 2.4 Evaluation of literature quality

The methodological quality of the included studies was independently evaluated by two researchers (J.Z. and Y.L.) using the NOS (27). The NOS evaluates studies across three domains: (1) selection of the study population (0–4 stars), (2) comparability between groups (0–2 stars), and (3) determination of exposure factors or outcome indicators (0–3 stars), yielding a maximum score of 9. Studies were categorized as high (≥7 stars), moderate (5–6 stars), or low (≤4 stars) quality. Disagreements were resolved through discussion, with unresolved issues adjudicated by a third researcher (D.B.).

### 2.5 Assessment criteria for OSA severity

The severity assessment of obstructive sleep apnea (OSA) is mainly based on the apnea-hypopnea index (AHI), representing the average number of apnea and hypopnea events per hour of sleep. According to the American Academy of Sleep Medicine (AASM) guidelines (28), the severity of OSA is classified as mild (AHI 5–14 events/hour), moderate (AHI 15–30 events/hour), and severe (AHI≥30 events/hour). The oxygen desaturation index (ODI), defined as the number of ≥3% blood oxygen saturation drops from baseline per hour (ODI 3%), served as an alternative metric. Given the well-established concordance between ODI and AHI metrics (29), when using ODI 3% ≥5 events/hour as the diagnostic threshold, its grading standard is consistent with that of AHI (30). If both AHI and ODI are reported in the study, the AHI grading is given priority. All included studies follow the original definition of OSA diagnosis and grading criteria.

### 2.6 Assessment criteria for the severity of DKD

The staging of DKD was based on the Kidney Disease: Improving Global Outcomes (KDIGO) 2012 clinical practice guidelines (31). The UACR is used as the core indicator, which is also the key basis for the early diagnosis of DKD. Here, microalbuminuria is defined as 30 mg/g<UACR ≤ 300 mg/g, indicating early renal impairment; while massive albuminuria is defined as UACR>300 mg/g, suggesting a significant decline in renal function.

### 2.7 Statistical analysis

All statistical analyses were performed using Review Manager 5.4.1. Effect sizes were estimated using odds ratios (OR) with 95% confidence intervals (CI) for dichotomous outcomes and mean differences (MD) with 95% CIs for continuous variables. Heterogeneity among studies was assessed using the I² statistic. An I² value ≤ 50% indicated low heterogeneity, and a fixed-effects model was applied; an I² value > 50% indicated substantial heterogeneity, and a random-effects model was used. Sensitivity analysis was conducted by sequentially removing each study to verify the robustness of the results and to explore potential sources of heterogeneity. Statistical significance was defined as P<0.05. To assess the robustness and generalizability of the results, 95% prediction intervals (PIs) were generated using R (v4.5.1) for meta-analyses with ≥3 studies, with results from both fixed- and random-effects models presented for comparison. Agreement in the effect direction between models suggested that the findings were insensitive to heterogeneity. Publication bias was evaluated using funnel plots and Egger’s test when ≥10 studies were included.

## 3 Result

### 3.1 Study selection and characteristics

**Figure 1:**
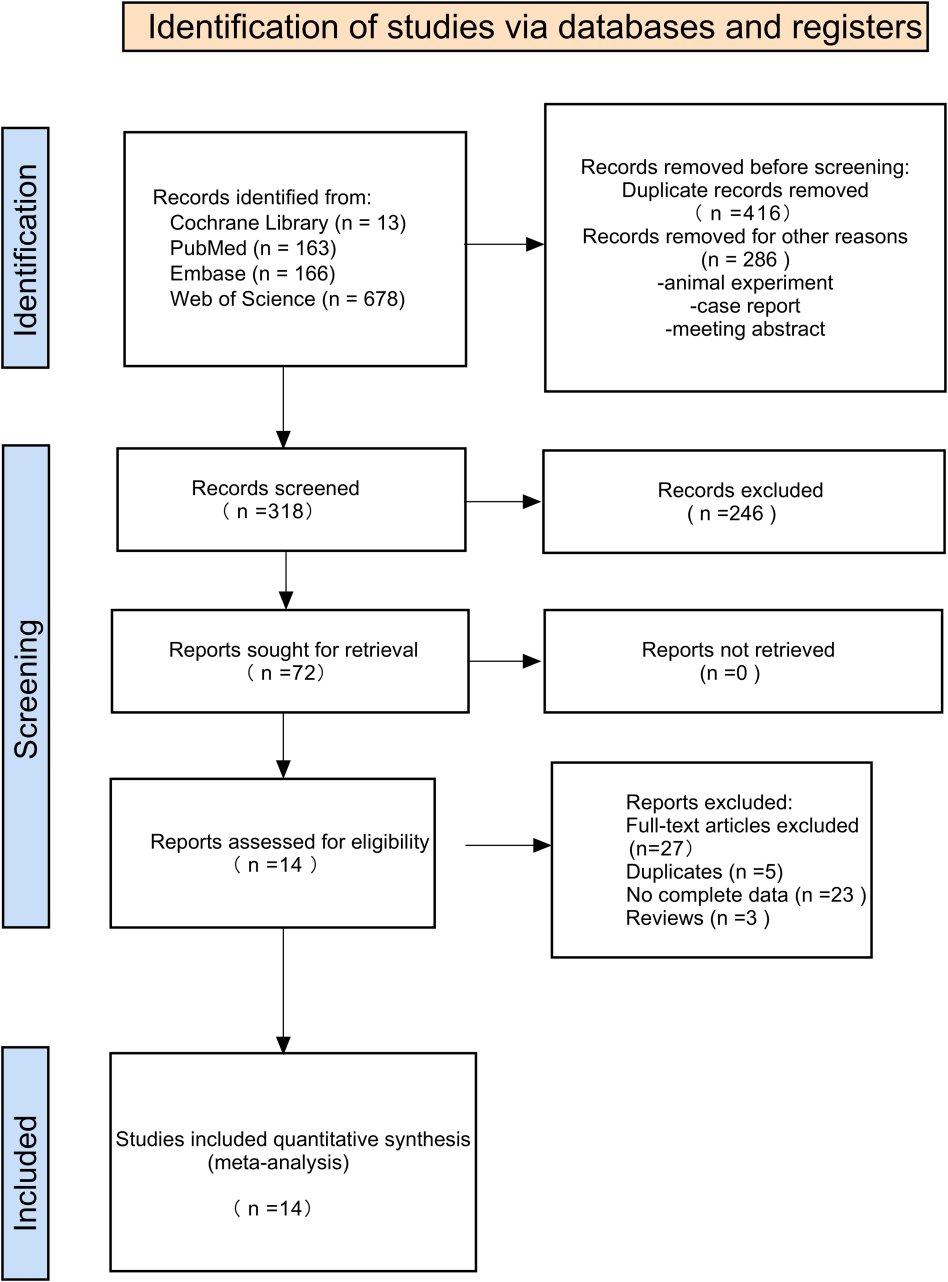
Flowchart of study selection and identification.

The study selection process followed the PRISMA guidelines (**Figure 1**). Initial database searches identified 1,020 records. After duplicate removal and preliminary screening, 318 articles underwent title and abstract assessment, of which 72 were selected for full-text review. Following a detailed evaluation against the inclusion and exclusion criteria, 14 studies were ultimately included in the systematic review and meta-analysis.

The baseline characteristics of the included studies are summarized in **Table 1**. These studies, published between 2012 and 2020, comprised 13 cross-sectional investigations and 1 prospective cohort study, collectively involving 5,316 diabetic patients with sample sizes ranging from 50 to 1,417. Among them, 10 studies (21, 32, 33, 35, 36, 39–43) (n = 3,517) investigated the prevalence of DKD in patients with and without OSA, while 3 studies (19, 34, 35) (n = 662) assessed the incidence of OSA in patients with and without DKD. One additional study (37) reported sleep parameters including AHI and oxygen saturation levels, but did not provide direct evidence regarding the association between OSA and DKD. The study populations were primarily from East Asia and Europe, including China (n = 6), Japan (n = 1), Denmark (n = 2), the United Kingdom (n = 3), Germany (n = 1), and France (n = 1). All studies diagnosed OSA objectively using polysomnography or portable monitoring devices. Most studies defined OSA as AHI ≥5 events/hour, while one study (21) used ODI 3% ≥5, and two others applied AHI thresholds of ≥15 and ≥10 events/hour (38, 40), respectively. DKD was diagnosed based on UACR ≥30 mg/g and/or eGFR <60 mL/min/1.73 m².

**Table 1:** Basic features of the included studies.

References	Country region	Case	Study designs	Study period	Sample size	Age	Male (n%)	BMI	Criteria for OSA	Criteria for DKD	Confounder
Dong et al., 2020 (32)	China	Hospital-based study	Cross-sectional study	September 2013-September 2018	322	56.8 ± 12.2	72.4	27.6 ± 3.7	PSG; AHI≥5 events/h	UACR≥30mg/g	SBP, DBP, BMI, AHI, duration of DM, hypertension, the use of ACEI/ARB
Zhang et al., 2016a (33)	China	Multi hospital-based study	Cross-sectional study	September 2012-February 2013	880	59. ± 12.7	55.6	25.1 ± 3.6	PSG; AHI≥5 events/h	UACR≥30mg/g/eGFR<60mL/min/1.73 m2	Gender, age, BMI, duration of DM
Yu et al., 2019 (34)	China	Hospital-based study	Cross-sectional study	November 2016-November 2017	109	52.77 ± 13.57	64.2	29.08 ± 4.36	PSG; AHI≥5 events/h	UACR≥30mg/g	Age, gender, and duration of DM, SBP, DBP
Xue et al., 2020 (35)	China	Hospital-based study	Cross-sectional study	December 2010–December 2018	463	/	63.1	/	PSG; AHI≥5 events/h	UACR≥30mg/g/eGFR<60mL/min/1.73 m2	Age and sex
Zhang et al., 2015 (36)	China	Multi hospital-based study	Cross-sectional study	February 2011–July 2011	472	54.5± 11.3	68	26.5 ± 3.9	PSG; AHI≥5 events/h	UACR≥30mg/g	Age and BMI
Zhang et al., 2016b (37)	China	Multi hospital-based study	Cross-sectional study	September 2012–February 2013	880	59.2 ± 12.7	55.6	25.1 ± 3.6	ApneaLink; AHI≥5 events/h	UACR≥30mg/g	Age, gender, duration of DM, BMI, hypertension, HbA1c
Stadler et al., 2017 (38)	Germany	Community-based study,	Cross-sectional study	2010-2014	679	65.6 ± 8.8	61	31.2 ± 5.5	PSG; AHI≥15 events/h	UACR≥30mg/g/eGFR<60mL/min/1.73 m	Gender, age, BMI, systolic blood pressure, duration of DM, HbA1c
Furukawa et al., 2013 (21)	Japan	Multi hospital-based study	Cross-sectional study	September 2009-December 2010	513	62.0 ± 10.5	56.9	25.2 ± 4.9	pulse oximetry;ODI 3% ≥ 5	UACR≥30mg/g	Gender, age, BMI, hypertension, hyperlipidemia
Banghoej et al., 2017 (39)	Denmark	Hospital-based study	Cross-sectional study	September 2013-December 2014	200	52 ± 15	68	25.3 ± 3.3	PSG; AHI≥5 events/h	UACR≥30mg/g	None
Meyer et al., 2019 (40)	France	Multi hospital-based study	Cross-sectional study	/	50	OSA 53.0 ± 12.4, Control 46.5 ± 11.9	52	OSA 28.2, Control 24.2	PSG; AHI≥10 events/h	Medical records	Age, BMI, duration of DM
Tahrani et al., 2012 (41)	UK	Multi hospital-based study	Cross-sectional study	/	234	57 ± 12	58	OSA 34.4, Control 30.2	PSG; AHI≥15 events/h	Medical records	Race, gender, duration of DM, BMI, hypertension, HbA1c
Leong et al., 2014 (19)	UK	Hospital-based study	Cross-sectional study	2009-2011	90	51 ± 10	43	46.8 ± 7.7	PSG; AHI≥5 events/h	eGFR<60mL/min/1.73 m2	Age, gender, BMI, insulin treatment, and drugs affecting the renin-angiotensin system.
Storgaard et al., 2014 (42)	Denmark	Hospital-based study	Cross-sectional study	September 2010-June 2011	200	59.6 ± 10.5	61	31.7 ± 6.7	PSG; AHI≥5 events/h	UACR≥30mg/g	None
Tahrani et al., 2013 (43)	UK	Multi hospital-based study	Cohort study	2009-2010	224	OSA 58.7, Control 54.8	OSA 97, Control 33	OSA+35.4, Control 31.6	PSG; AHI≥5 events/h	eGFR<60mL/min/1.73 m2	Gender, race, age, duration of DM, BMI, hypertension

### 3.2 Quality assessment of included studies

The methodological quality of the included observational studies was appraised using the NOS. As summarized in **Table 2**, the NOS scores ranged from 6 to 9, indicating that all studies were of moderate to high quality. This reflects the moderate to low risk of bias in the included studies, and no studies were excluded due to low quality (scores < 5).

**Table 2:** Newcastle–Ottawa scale of the included studies.

References	Selection	Comparability	Exposure	Scores	Quality
Dong et al., 2020 (32)	***	*	**	6	Moderate
Zhang et al., 2016a (33)	***	**	***	8	High
Yu et al., 2019 (34)	***	**	**	7	High
Xue et al., 2020 (35)	***	**	**	7	High
Zhang et al., 2015 (36)	***	**	**	7	High
Zhang et al., 2016b (37)	***	**	***	8	High
Stadler et al., 2017 (38)	****	**	***	9	High
Furukawa et al., 2013 (21)	***	**	***	8	High
Banghoej et al.,2017 (39)	***	*	***	7	High
Meyer et al., 2019 (40)	**	**	**	6	Moderate
Tahrani et al., 2012 (41)	**	**	***	7	High
Leong et al., 2014 (19)	**	*	***	6	Moderate
Storgaard et al., 2014) (42)	***	**	***	8	High
Tahrani et al., 2013 (43)	**	**	***	7	High

* represents the score for each domain of the NOS for assessing the quality of observational studies.

### 3.3 Meta-analysis results of the association between OSA and DKD

#### 3.3.1 Correlation between OSA and the incidence of DKD

**Figure 2:**
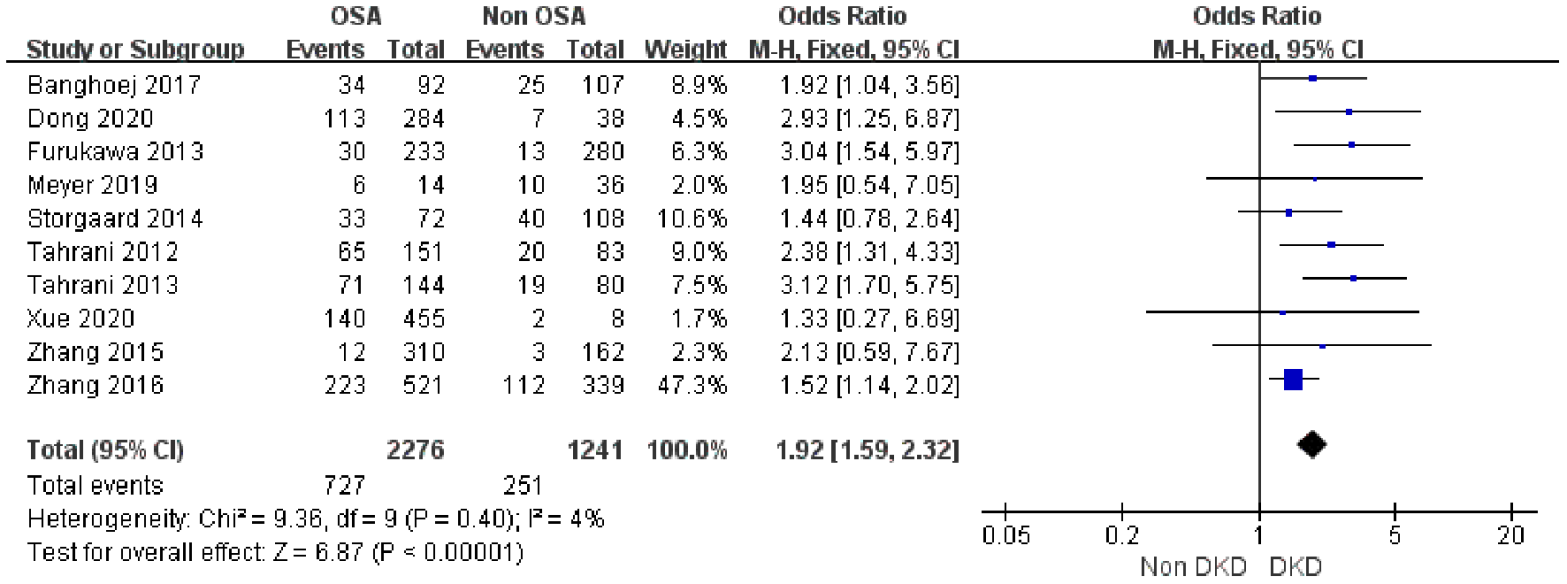
Forest plot of the association between OSA and incident DKD.

Ten studies (21, 32, 33, 35, 36, 39–43) involving 3,517 subjects were included in the analysis of the association between OSA and DKD incidence. The study population comprised 2,276 patients in the OSA group (727 with DKD) and 1,241 in the non-OSA group (251 with DKD). Given the low heterogeneity (I² = 4%), a fixed-effect model was employed. Meta-analysis demonstrated a significant association between OSA and DKD (OR = 1.92, 95% CI: 1.59 - 2.32, 95%PI:1.22-3.39, P < 0.0001) (**Figure 2**).

#### 3.3.2 Association between mild to moderate/severe OSA and DKD

**Figure 3:**
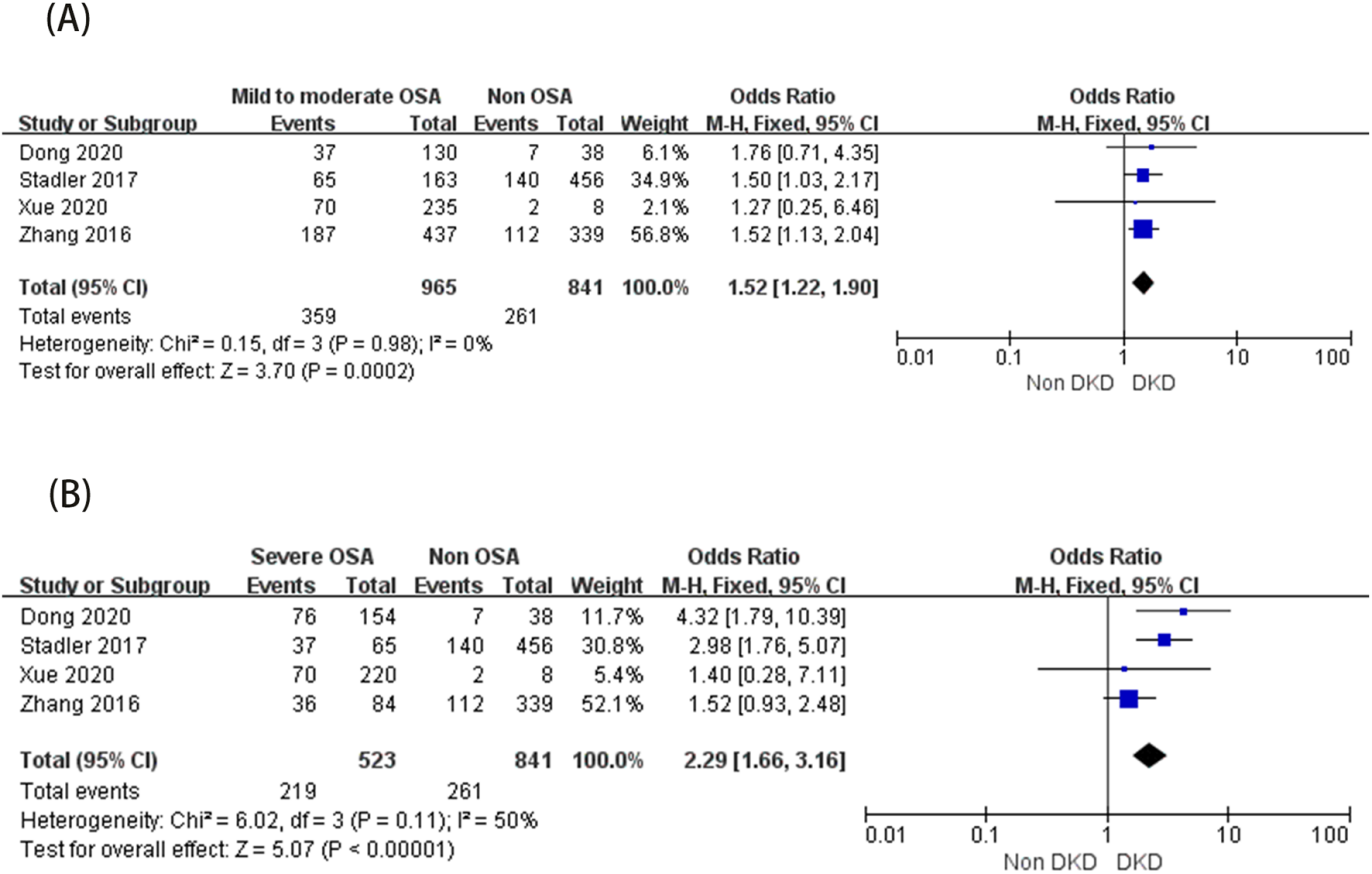
**(A)** Forest plot of the association between mild to moderate and DKD. **(B)** Forest plot of the association between severe OSA and DKD.

A meta-analysis was conducted by dividing the subjects into two groups based on the severity of OSA: mild to moderate OSA (AHI 5–30 times/hour) and severe OSA (AHI ≥ 30 times/hour). All three studies included in this specific analysis used AHI criteria for OSA severity classification. The results showed that mild to moderate OSA was significantly associated with the risk of DKD (OR = 1.52, 95% CI 1.22–1.90, 95% PI:1.06-2.18, P = 0.0002), and there was no heterogeneity among the studies (I² = 0%) (**Figures 3A**). More importantly, the risk of DKD in patients with severe OSA was further increased, with a combined OR value of 2.29 (95% CI: 1.66–3.16, 95% PI: 0.56-10.07, P < 0.00001). This group had moderate heterogeneity (I² = 50%), and a fixed effects model was adopted. (**Figures 3B**) Sensitivity analysis revealed that the heterogeneity was primarily derived from the study by Zhang et al. (33), which employed a multi-center design distinct from the single-center designs of the other studies. Excluding this study eliminated heterogeneity (I² = 0%; see **Supplementary Figure 1**).

#### 3.3.3 Association between OSA and eGFR

**Figure 4:**
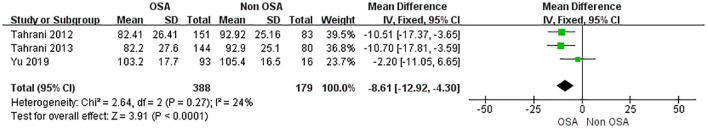
Forest plot of the association between OSA and eGFR.

Three studies (34, 41, 43)were included in the analysis of the association between OSA and eGFR. The results showed that the eGFR level of OSA patients was significantly lower than that of non-OSA patients (MD = -8.61, 95% CI: -12.92- -4.30, 95% PI:-20.62-3.58, P < 0.0001). The heterogeneity among the studies was low (I² = 24%), supporting a fixed-effects model approach (**Figure 4**).

### 3.4 Meta-analysis results of the association between DKD and OSA

#### 3.4.1 Correlation between DKD and the Incidence of OSA

**Figure 5:**
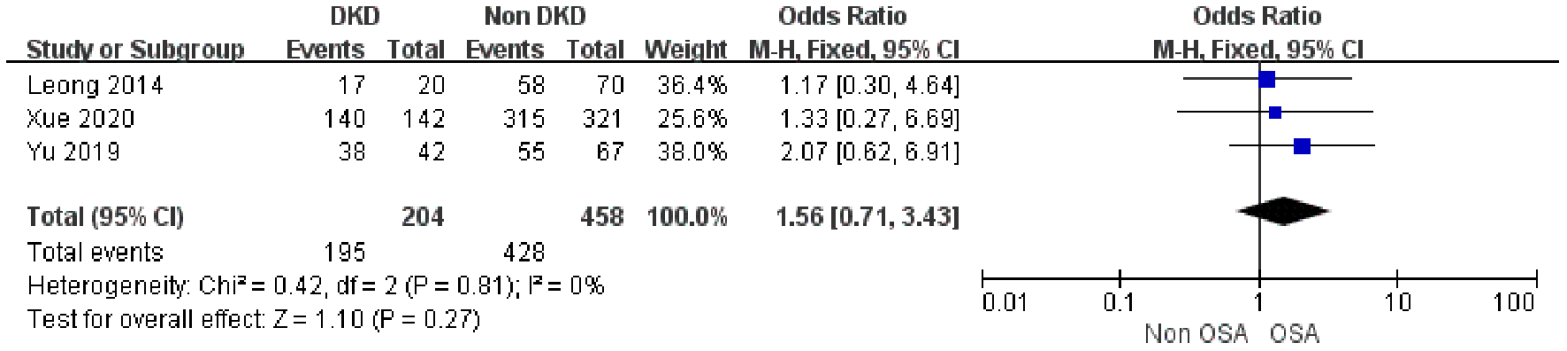
Forest plot of the association between DKD and incident OSA.

Three studies (19, 34, 35) involving 662 participants were included in the analysis of the association between DKD and OSA incidence. The study population comprised 204 patients in the DKD group (195 with OSA) and 458 in the non-DKD group (428 with OSA). No significant heterogeneity was detected (I² = 0%), supporting the use of a fixed-effect model. Meta-analysis revealed no statistically significant association between DKD and OSA incidence (OR = 1.56, 95% CI: 0.71 - 3.43, 95% PI: 0.27-8.75, P = 0.27) (**Figure 5**).

#### 3.4.2 Association between severity of proteinuria in DKD and AHI

**Figure 6:**
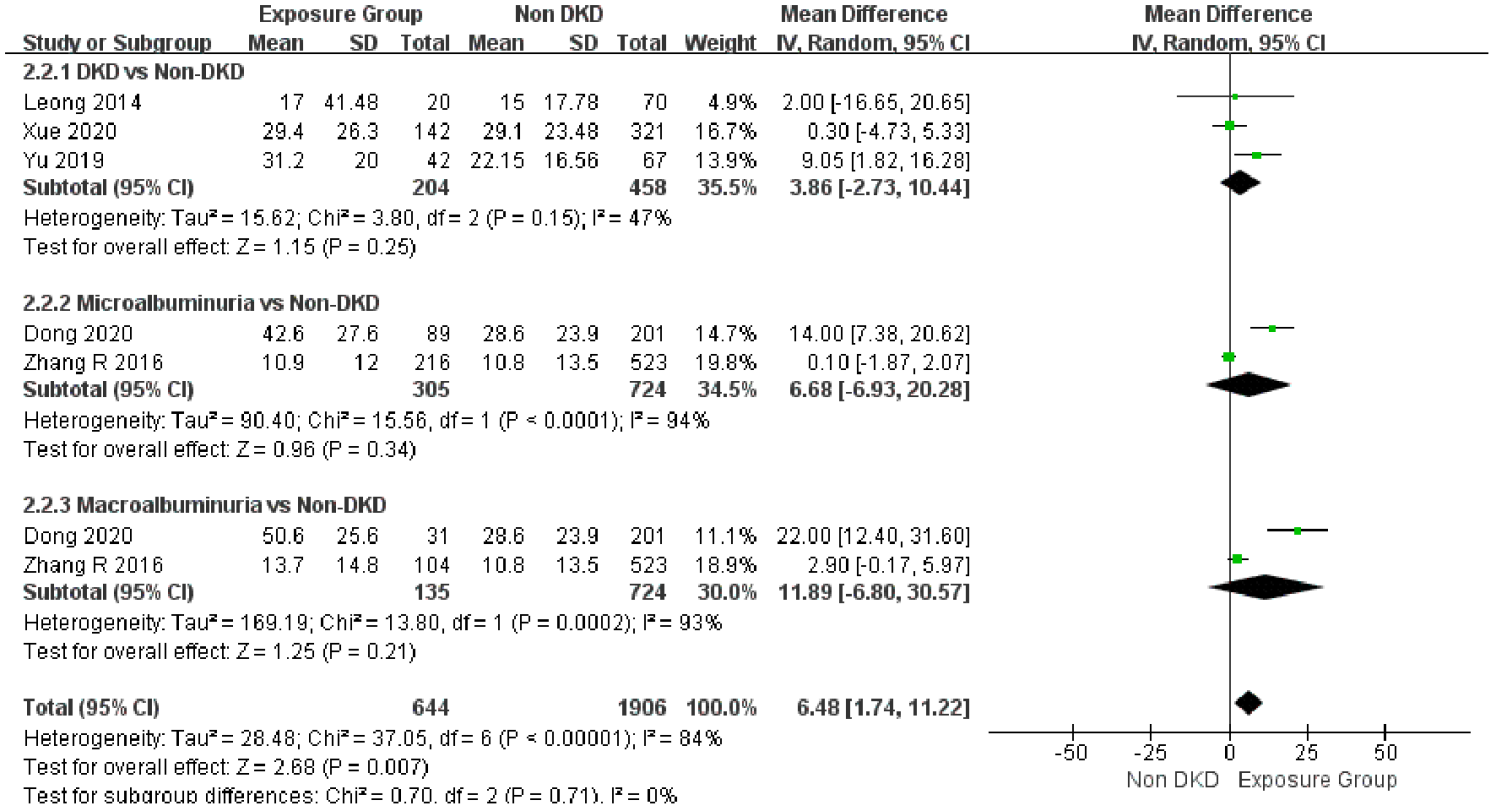
Forest plot of the association between proteinuria severity and AHI in DKD.

A pooled analysis of five studies (19, 32, 34, 35, 37) demonstrated that patients with DKD had significantly higher AHI levels than non-DND patients (MD = 6.48, 95% CI: 1.74 - 11.22, 95% PI: -12.24-25.93, P = 0.007), though substantial heterogeneity was observed (I² = 84%). To explore the source of heterogeneity, a sensitivity analysis was conducted. After excluding the study by Yu et al (34), targeting a specific obese T2 DM population, the heterogeneity significantly decreased (I² = 0%; see **Supplementary Figure 2**), suggesting that this study might be the main source of heterogeneity. Subgroup analysis revealed that the increase in AHI was not statistically significant in patients with diagnosed DKD (MD = 3.86, P = 0.25). Similarly, no significant differences were observed in those with microalbuminuria (MD = 6.68, P = 0.34) or macroalbuminuria (MD = 11.89, P = 0.21), with both subgroups showing considerable heterogeneity (I² > 90%) (**Figure 6**).

#### 3.4.3 Association between the severity of proteinuria in DKD and L-SaO_2_

A pooled analysis of five studies (19, 32, 34, 35, 37) revealed that DKD patients showed lower average L-SaO^2^ compared with non-DKD patients (95% CI: -1.27 - 0.35, 95% PI: -4.25-2.06), but the difference was not statistically significant (P = 0.27), and there was moderate heterogeneity among the studies (I² = 48%). Subgroup analysis revealed that the reduction in L-SaO_2_ in diagnosed DKD patients (MD = -1.31, P = 0.16), patients with microalbuminuria (MD = 0.06, P = 0.91), and patients with massive albuminuria (MD = -0.94, P = 0.26) did not reach statistical significance. Among them, the subgroup with massive albuminuria had high heterogeneity (I² = 81%), and no significant differences were found across proteinuria severity subgroups (P = 0.36) (**Supplementary Figure 3**).

#### 3.4.4 Association between the severity of proteinuria in DKD and M-SaO_2_

**Figure 7:**
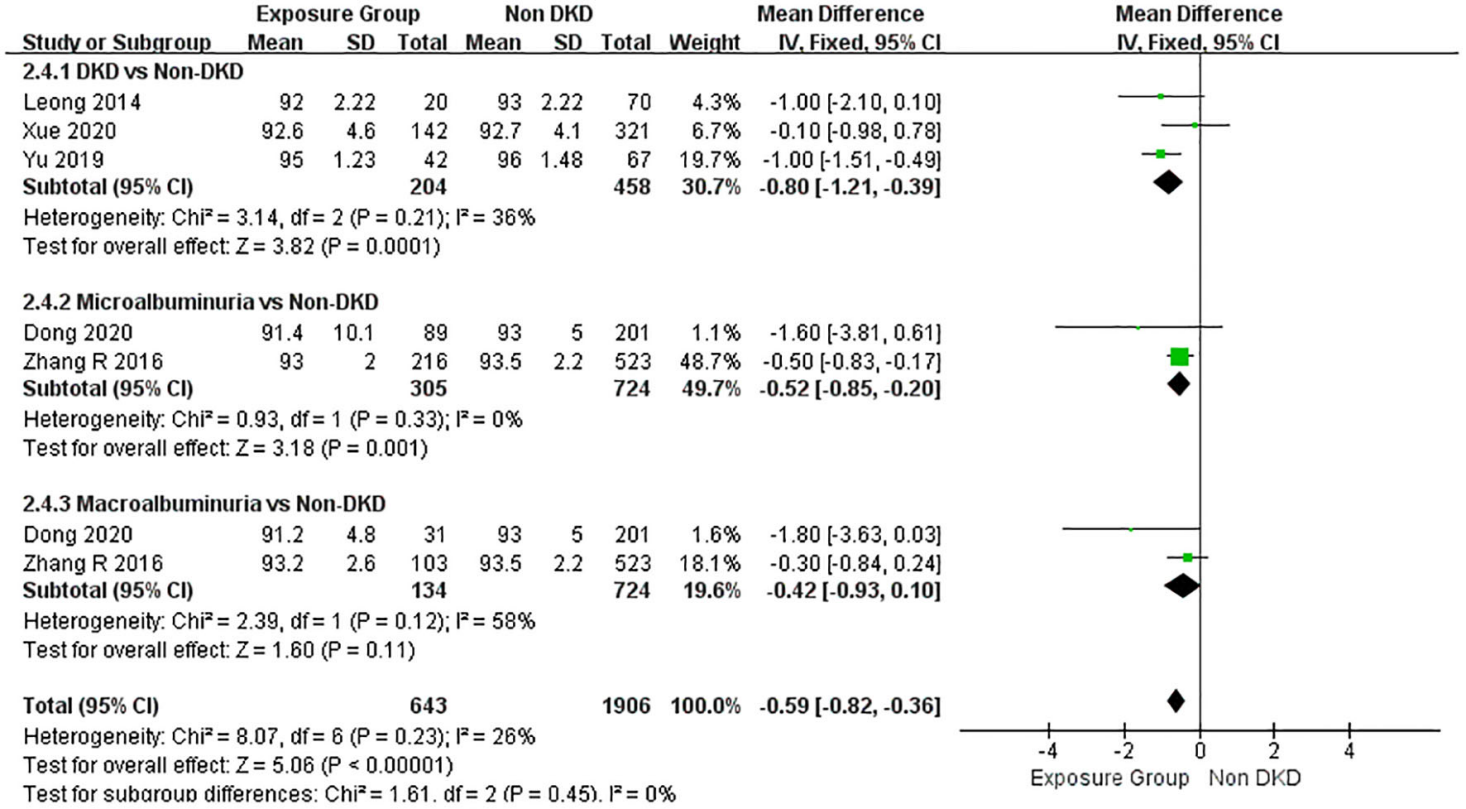
Forest plot of the association between proteinuria severity and M-SaO_2_ in DKD.

A pooled analysis of five studies (19, 32, 34, 35, 37) demonstrated significantly lower M-SaO^2^ levels in DKD patients compared with non-DKD controls (MD = -0.59, 95% CI: -0.82 - -0.36, 95% PI: 1.22- -0.02, P < 0.00001), with low heterogeneity among studies (I² = 26%). Subgroup analysis confirmed this negative association across proteinuria severity strata: significant M-SaO^2^ reductions were observed in patients with diagnosed DKD (MD = -0.80, P = 0.0001) and microalbuminuria (MD = -0.52, P = 0.001). A non-significant downward trend was noted in macroalbuminuria patients (MD = -0.42%, P = 0.11), but it did not reach statistical significance. There was no statistically significant difference among the subgroups (P = 0.45) (**Figure 7**).

### 3.5 Heterogeneity analysis

In all meta-analyses of the bidirectional OSA-DKD association, the fixed-effect and random-effects models produced concordant results in effect direction (**Supplementary Figures S5-S12**). This indicates that no substantial heterogeneity was present in the respective analyses.

### 3.6 Publication bias assessment

**Figure 8:**
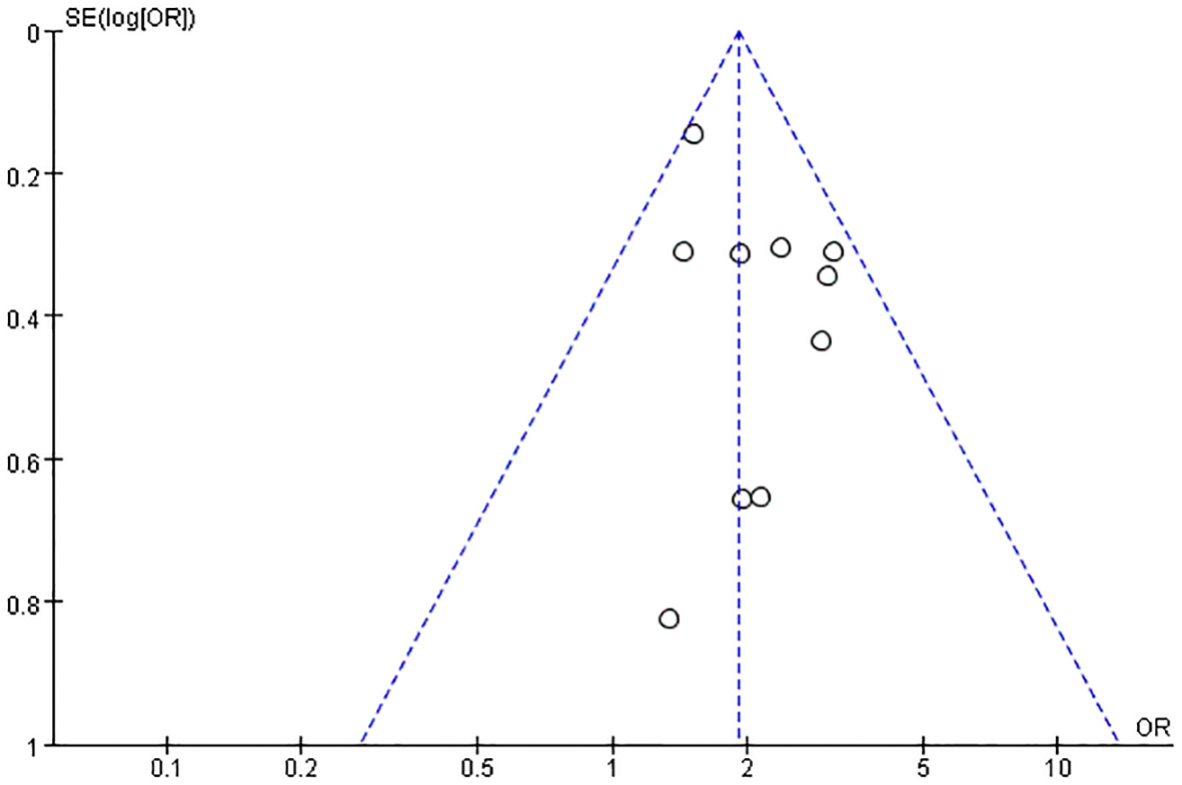
Funnel plot of the incidence rate of DKD in OSA.

Publication bias was assessed for the association between OSA and DKD (10 studies) using funnel plot inspection and Egger’s regression test. The funnel plot showed general symmetry (**Figure 8**), and Egger’s test indicated no significant bias (P = 0.189). These consistent results from two complementary methods suggest minimal publication bias in our findings.

### 3.7 Sensitivity analysis

To evaluate the robustness of the pooled results, we performed sensitivity analyses by sequentially excluding individual studies. The direction and statistical significance of the main effects remained consistent throughout all sensitivity tests, supporting the reliability of our conclusions. In the analysis of “Association between the severity of proteinuria in DKD and AHI”, excluding the study by Yu et al. (34) reduced heterogeneity from 84% to 0% without altering the direction or significance of the pooled effect, identifying this study as the primary source of heterogeneity. Similarly, for the severe OSA and DKD analysis, the observed heterogeneity (I² = 50%) was entirely attributable to the multi-center study by Zhang et al (33), as shown by its elimination upon the study’s exclusion (I² = 0%). Further evaluation of the relationship between proteinuria severity and nocturnal oxygen parameters indicated that the association between DKD and M−SaO^2^ was the most robust. After eliminating any study, the combined MD ranged from –0.49 to –0.88, and all remained statistically significant. For AHI, the association with DKD persisted after excluding most studies; however, removal of Dong et al (32)led to loss of statistical significance (MD = 1.93, P = 0.14); In contrast, the association between DKD and L−SaO^2^ was highly sensitive to the study by Zhang et al (37): exclusion changed the result from non−significant to significant (MD = –1.95, P = 0.01). These results suggest that the differences in effect sizes among some cross-subgroup studies might be an important source of heterogeneity. Complete sensitivity analysis results are provided in **Supplementary Table S3-10**.

### 3.8 Valuation of evidence quality

We assessed evidence quality using the GRADE tool, which categorizes evidence as high, moderate, low, or very low based on five domains: risk of bias, inconsistency, indirectness, imprecision, and publication bias. The presence of any of these limitations lowers the certainty of evidence. In this study, the evidence for the association between OSA and DKD was generally of high or moderate quality. In contrast, the reverse association of DKD with OSA was predominantly moderate to low. Complete GRADE profiles are provided in **Supplementary Figure S4**.

## 4 Discussion

This study, integrating evidence from 14 moderate-to-high quality observational studies (5,316 participants), provides the first systematic evidence of a significant bidirectional association between OSA and DKD. This relationship demonstrates a clear dose-response pattern and indicator specificity, offering a key evidence base for clinical co-management of these conditions.

In the positive association between OSA and DKD, we found that the risk of DKD in OSA patients was significantly higher than that in non-OSA patients (OR = 1.92), and there was a clear dose-response relationship. Specifically, severe OSA conferred a nearly 60% higher DKD risk (OR = 2.37) compared to mild OSA (OR = 1.52), supporting OSA severity as an independent risk stratifier for DKD. Further analysis of core renal function indicators revealed that the eGFR level of OSA patients was significantly lower (MD = -8.61). This finding was consistent with the results of Tahrani’s (41) prospective study and further confirmed the negative impact of OSA on renal function progression. This is further corroborated by Nicholl et al (44), who reported significantly higher renal failure risk in patients with coexisting OSA and DKD.

Analysis of the reverse association (DKD to OSA) revealed a distinct phenotype: while OSA prevalence did not significantly differ between DKD and non-DKD groups (OR = 1.56, P = 0.27), DKD patients demonstrated significantly higher AHI and lower M-SaO^2^, particularly with progressive proteinuria. This “unchanged prevalence but increased severity” pattern suggests DKD may exacerbate pre-existing OSA through pathological processes that amplify hypoxic burden. Sensitivity analyses supported the robustness of these findings, though residual heterogeneity was observed, primarily attributable to variations in population characteristics (e.g., obesity prevalence), OSA diagnostic criteria, and confounding control. Notably, the association between DKD and M-SaO^2^ proved more consistent than those with AHI or L-SaO^2^, suggesting M-SaO^2^ may be a more reliable marker of OSA severity in DKD patients—a finding consistent with Zhang et al.’s (37)report of a close M-SaO^2^–microalbuminuria correlation.

In the DKD-AHI analysis, high heterogeneity (I² = 84%) was largely driven by Yu et al (34), which focused on an obese T2DM population, identifying BMI as a key confounder. Similarly, moderate heterogeneity (I² = 50%) in the severe OSA-DKD analysis was traced to Zhang et al.’s (33)multi-center design. Furthermore, methodological variations significantly influenced the effect estimates. For instance, Dong et al (32) employed a single-center inpatient design with a higher proportion of obese participants and used polysomnography (PSG), which exhibits greater sensitivity in detecting hypopnea events. Consequently, their reported AHI values were substantially elevated in the macroalbuminuria subgroup. In contrast, Zhang (37)’s multi-center study recruited a more heterogeneous population and relied on a portable monitor (ApneaLink) with a less stringent hypopnea threshold, potentially underestimating mild hypopnea events. Thereby attenuating the actual L-SaO^2^ differences between DKD and non-DKD groups and potentially contributing to the non-significant findings in the original analysis.

The non-significant association between DKD and OSA prevalence requires careful interpretation. First, with only three available studies, the analysis was underpowered due to limited sample size. Second, a threshold effect may exist whereby increased OSA prevalence may become apparent only in advanced DKD stages (eGFR <30 mL/min/1.73m²), where metabolic disturbances and fluid retention are more pronounced; however, the current analysis lacked stratification by renal function severity. Third, residual confounding from factors such as diuretic use (potentially exacerbating upper airway dryness) and sleep duration (intrinsically linked to OSA diagnosis) in some original studies may have obscured the true association. Together, these factors may have led to an underestimation of the true effect size. Thus, future investigations with larger cohorts and standardized protocols are warranted to clarify this relationship.

Intermittent hypoxia (IH) and sleep fragmentation, as the core pathological features of OSA, contribute to the development and progression of diabetic kidney disease (DKD) through complex cascade mechanisms. Specifically, IH mimics ischemia-reperfusion injury (45), thereby activating multiple pathways, including xanthine oxidase, NADPH oxidase, and the mitochondrial electron transport chain. This process leads to substantial overproduction of reactive oxygen species (ROS) that disrupts the systemic oxidative-antioxidant balance (46, 47). These excessive ROS not only directly promote mesangial cell hypertrophy, podocyte apoptosis, and enhanced permeability of the glomerular basement membrane, thereby initiating early kidney damage (48), but also further activate the nuclear factor-κB (NF-κB) signaling pathway. Mechanistically, IH-induced oxygen deprivation inhibits IκB kinase hydroxylation, facilitating the degradation of NF-κB inhibitors and enabling NF-κB to translocate to the nucleus and bind to the promoters of target genes (49, 50), thereby regulating the expression of pro-inflammatory factors such as IL-1, IL-6, IL-18, and tumor necrosis factor-α (TNF-α). This cascade simultaneously promotes the accumulation of advanced glycation end products (AGEs) and recruits M1-type macrophages into the renal interstitium, stimulating fibronectin production and fibroblast proliferation, ultimately accelerating renal fibrosis (51, 52). This mechanistic framework aligns with previous reports indicating that inflammatory markers mediate sleep deprivation–induced DKD progression, thereby refining our understanding of OSA-associated kidney injury (53).

In addition to the core pathways involving oxidative stress and inflammation, OSA contributes to renal impairment through several additional synergistic mechanisms. First, IH activates hypoxia-inducible factor-1α (HIF-1α) (54), inducing the expression of vascular endothelial growth factor (VEGF) (55). While this adaptation may temporarily compensate for tissue hypoxia, sustained HIF-1α activation promotes pathological glomerular angiogenesis and basement membrane thickening, accelerating renal structural damage. Second, sleep fragmentation induces persistent sympathetic activation (34), which synergizes with intermittent hypoxia to stimulate the RAAS (56). This in turn enhances glomerular hyperfiltration and hyperperfusion, driving tubulointerstitial injury and progressive eGFR decline—a correlation clinically validated between sleep fragmentation severity and eGFR deterioration rates (57). Furthermore, sleep deprivation and circadian disruption associated with OSA promote metabolic dysregulation, enhancing hepatic glucose production and gluconeogenesis, which exacerbate hyperglycemia and insulin resistance (58), along with SREBP1-mediated lipogenesis. These metabolic abnormalities further amplify microvascular injury via the AGE/RAGE axis and protein kinase C (PKC) signaling (59). Of particular interest, recent evidence indicates that OSA-related sleep disturbances elevate plasma levels of fibroblast growth factor 23 (FGF-23), which is an independent risk factor for CKD progression (60, 61). Thus, FGF-23 may represent a novel molecular link between OSA and DKD, potentially explaining the dose–response relationship between OSA severity and DKD risk.

The impact of DKD on OSA manifests primarily as exacerbation of pre-existing conditions rather than induction of new cases, involving both structural remodeling and functional dysregulation. At the structural level, uremic toxin accumulation during DKD progression contributes to peripheral neuropathy, reducing upper airway dilator muscle tone and promoting soft tissue edema (62). Critically, the “medullary overnight fluid shift” (63) phenomenon, wherein fluid retained due to renal dysfunction redistributes to the pharynx and larynx during recumbency, narrows the upper airway and elevates the risk of airway collapse (64). Anatomic evidence supports this mechanism, revealing significant pharyngeal narrowing in end-stage renal disease patients (18), which provides a morphological basis for DKD-mediated OSA exacerbation. Functionally, metabolic acidosis and altered chemoreceptor sensitivity drive respiratory dysregulation (62). As renal function deteriorates, hydrogen ion accumulation chronically stimulates peripheral chemoreceptors, initially enhancing ventilation compensatorily but ultimately blunting respiratory center sensitivity to hypoxia/hypercapnia and promoting hypoventilation (65). Notably, OSA patients exhibit both peripheral and central chemoreceptor hyperreactivity, a dysfunction linked to uremic toxins and acidosis (66), further destabilizing respiratory control.

Furthermore, several comorbid conditions commonly associated with DKD contribute synergistically to disease progression. Anemia compromises oxygen-carrying capacity, potentiating IH-induced respiratory suppression (67). Meanwhile, hypertension and obesity—shared risk factors for both OSA and DKD—promote airway collapsibility through increased airway resistance and peripharyngeal fat deposition (68). Together, these factors establish a vicious “DKD-OSA-renal injury” cycle whereby DKD-induced airway structural changes and ventilatory control abnormalities elevate AHI and hypoxic burden, which in turn drives further renal impairment via oxidative stress, inflammatory pathways, and other previously described mechanisms, collectively accelerating disease progression.

This study provides new insights through systematic evaluation of the bidirectional association between OSA and DKD, a critical perspective overlooked in previous unidirectional studies including Leong et al.’s 2016 meta-analysis (69). Our findings demonstrate that their interaction is characterized by mutual exacerbation rather than unidirectional effects. Compared with Leong’s work, key advances include: systematic validation of the reverse association between DKD and OSA severity, addressing a fundamental gap identified but unresolved in previous research; integration of larger medium-to-high quality samples with specific stratified analyses of OSA severity and DKD progression, confirming severe OSA as a high-risk factor for DKD and the superior diagnostic sensitivity of M-SaO^2^ over L-SaO^2^. Rigorous sensitivity analyses further clarified heterogeneity sources and the impact of study design and population characteristics, filling important gaps in understanding the mechanisms and clinical implications of OSA-DKD interactions.

## 5 Limitations

This study has several limitations that should be considered (1): Only English-language studies were included, and grey literature or unpublished works were not searched, which may introduce selection bias. (2) The predominance of cross-sectional designs among the included studies enables the demonstration of association but prevents the establishment of causality. (3) Despite established consistency between ODI and AHI metrics, variability in diagnostic criteria for OSA and definitions of DKD across studies remains a concern. (4) Residual confounding from unmeasured factors may influence the accuracy of the pooled effect estimates. (5) The analysis of the reverse association included fewer studies, restricting statistical power. (6) Although sensitivity analyses were performed, subgroup analyses based on key clinical characteristics were not feasible due to limited data availability. (7) Although trends were observed between proteinuria severity and OSA parameters, the available data did not permit detailed dose-response analysis, limiting insight into the dynamic effects of DKD progression on OSA. (8) The absence of dynamic measures such as cumulative time spent with SpO_2_ < 90% (CT90%) and annual eGFR decline rate precludes assessment of the temporal aspects of this relationship. (9) The latest included studies were up to 2020, lacking recent data.

## 6 Conclusion

This study demonstrates a clear bidirectional relationship between OSA and DKD: OSA significantly elevates the risk of DKD with a distinct dose-response relationship, where severe OSA confers the highest risk; while DKD does not increase OSA prevalence, it markedly exacerbates disease severity, evidenced by elevated AHI and reduced M-SaO^2^. These findings support routine screening for OSA in diabetic patients with eGFR <60 mL/min/1.73 m² or UACR ≥30 mg/g, and regular renal function monitoring in OSA patients, particularly those with severe disease (AHI ≥30 events/hour). Future research should focus on elucidating shared hypoxia-inflammation mechanisms and validating causality through prospective studies to optimize integrated management of these comorbid conditions.

## References

[1] Young T Peppard PE Gottlieb DJ . Epidemiology of obstructive sleep apnea: A population health perspective. Am J Respir Crit Care Med. (2002) 165:1217–39. doi: 10.1164/rccm.2109080, PMID: 11991871

[2] McArdle N Hillman D Beilin L Watts G . Metabolic risk factors for vascular disease in obstructive sleep apnea: A matched controlled study. Am J Respir Crit Care Med. (2007) 175:190–5. doi: 10.1164/rccm.200602-270OC, PMID: 17068329

[3] Peppard PE Young T Barnet JH Palta M Hagen EW Hla KM . Increased prevalence of sleep-disordered breathing in adults. Am J Epidemiol. (2013) 177:1006–14. doi: 10.1093/aje/kws342, PMID: 23589584 PMC3639722

[4] Heinzer R Vat S Marques-Vidal P Marti-Soler H Andries D Tobback N . Prevalence of sleep-disordered breathing in the general population: the hypnolaus study. Lancet Respir Med. (2015) 3:310–8. doi: 10.1016/S2213-2600(15)00043-0, PMID: 25682233 PMC4404207

[5] Young T Peppard PE Taheri S . Excess weight and sleep-disordered breathing. J Of Appl Physiol. (2005) 99:1592–9. doi: 10.1152/japplphysiol.00587.2005, PMID: 16160020

[6] Pepperell JC Davies RJ Stradling JR . Systemic hypertension and obstructive sleep apnoea. Sleep Med Rev. (2002) 6:157–73. doi: 10.1053/smrv.2001.0189, PMID: 12531119

[7] Cheng H-T Xu X Lim PS Hung K-Y . Worldwide epidemiology of diabetes-related end-stage renal disease, 2000–2015. Diabetes Care. (2021) 44:89–97. doi: 10.2337/dc20-1913, PMID: 33203706

[8] Cole JB Florez JC . Genetics of diabetes mellitus and diabetes complications. Nat Rev Nephrol. (2020) 16:377–90. doi: 10.1038/s41581-020-0278-5, PMID: 32398868 PMC9639302

[9] Tenda ED Henrina J Cha JH Triono MR Putri EA Aristy DJ . Obstructive sleep apnea: overlooked comorbidity in patients with diabetes. World J Diabetes. (2024) 15:1448. doi: 10.4239/wjd.v15.i7.1448, PMID: 39099813 PMC11292334

[10] Somers VK Dyken ME Clary MP Abboud FM . Sympathetic neural mechanisms in obstructive sleep apnea. J Clin Invest. (1995) 96:1897–904. doi: 10.1172/JCI118235, PMID: 7560081 PMC185826

[11] Yan SF Ramasamy R Schmidt AM . The receptor for advanced glycation endproducts (Rage) and cardiovascular disease. Expert Rev In Mol Med. (2009) 11:e9. doi: 10.1017/S146239940900101X, PMID: 19278572 PMC2670065

[12] Reutrakul S Mokhlesi B . Obstructive sleep apnea and diabetes a state of the art review. CHEST. (2017) 152:1070–86. doi: 10.1016/j.chest.2017.05.009, PMID: 28527878 PMC5812754

[13] Lavie L . Oxidative stress in obstructive sleep apnea and intermittent hypoxia - revisited - the bad ugly and good: implications to the heart and brain. Sleep Med Rev. (2015) 20:27–45. doi: 10.1016/j.smrv.2014.07.003, PMID: 25155182

[14] Hakim F Gozal D Kheirandish-Gozal L . Sympathetic and catecholaminergic alterations in sleep apnea with particular emphasis on children. Front Neurol. (2012) 3:7. doi: 10.3389/fneur.2012.00007, PMID: 22319509 PMC3268184

[15] Tucker PS Dalbo VJ Han T Kingsley MI . Clinical and research markers of oxidative stress in chronic kidney disease. Biomarkers: Biochem Indic Expos Res Susceptibil to Chem. (2013) 18:103–15. doi: 10.3109/1354750x.2012.749302, PMID: 23339563

[16] Sun W Yin X Wang Y Tan Y Cai L Wang B . Intermittent hypoxia-induced renal antioxidants and oxidative damage in male mice: hormetic dose response. Dose-response: Publ Int Hormesis Soc. (2012) 11:385–400. doi: 10.2203/dose-response.12-027.Cai, PMID: 23983666 PMC3748850

[17] Chiu KL Ryan CM Shiota S Ruttanaumpawan P Arzt M Haight JS . Fluid shift by lower body positive pressure increases pharyngeal resistance in healthy subjects. Am J Respir Crit Care Med. (2006) 174:1378–83. doi: 10.1164/rccm.200607-927OC, PMID: 16998093

[18] Beecroft JM Hoffstein V Pierratos A Chan CT McFarlane PA Hanly PJ . Pharyngeal narrowing in end-stage renal disease: implications for obstructive sleep apnoea. Eur Respir J. (2007) 30:965–71. doi: 10.1183/09031936.00161906, PMID: 17626107

[19] Leong WB Nolen M Thomas GN Adab P Banerjee D Taheri S . The impact of hypoxemia on nephropathy in extremely obese patients with type 2 diabetes mellitus. J Of Clin Sleep Med. (2014) 10:773–8. doi: 10.5664/jcsm.3870, PMID: 25024655 PMC4067441

[20] Chou YT Lee PH Yang CT Lin CL Veasey S Chuang LP . Obstructive sleep apnoea: A stand-alone risk factor for chronic kidney disease. Nephrol Dialysis Transplant. (2011) 26:2244–50. doi: 10.1093/ndt/gfq821, PMID: 21317406

[21] Furukawa S Saito I Yamamoto S Miyake T Ueda T Niiya T . Nocturnal intermittent hypoxia as an associated risk factor for microalbuminuria in Japanese patients with type 2 diabetes mellitus. Eur J Endocrinol. (2013) 169:239–46. doi: 10.1530/EJE-13-0086%JEuropeanJournalofEndocrinology, PMID: 23704715

[22] Buyukaydin B Akkoyunlu ME Kazancioglu R Karakose F Ozcelik HK Erkoc R . The effect of sleep apnea syndrome on the development of diabetic nephropathy in patients with type 2 diabetes. Diabetes Res Clin Pract. (2012) 98:140–3. doi: 10.1016/j.diabres.2012.07.007, PMID: 22906637

[23] Zhang X Tan R Lam WC Yao L Wang X Cheng CW . Prisma (Preferred reporting items for systematic reviews and meta-analyses) extension for chinese herbal medicines 2020 (Prisma-chm 2020). Am J Chin Med. (2020) 48:1279–313. doi: 10.1142/S0192415X20500639, PMID: 32907365

[24] Stroup DF Berlin JA Morton SC Olkin I Williamson GD Rennie D . Meta-analysis of observational studies in epidemiology: A proposal for reporting. Jama. (2000) 283:2008–12. doi: 10.1001/jama.283.15.2008, PMID: 10789670

[25] Kushida CA Littner MR Morgenthaler T Alessi CA Bailey D Coleman J Jr. . Practice parameters for the indications for polysomnography and related procedures: an update for 2005. Sleep. (2005) 28:499–521. doi: 10.1093/sleep/28.4.499, PMID: 16171294

[26] Collop NA Anderson WM Boehlecke B Claman D Goldberg R Gottlieb DJ . Clinical guidelines for the use of unattended portable monitors in the diagnosis of obstructive sleep apnea in adult patients. Portable monitoring task force of the american academy of sleep medicine. J Clin sleep Med: JCSM: Off Publ Am Acad Sleep Med. (2007) 3:737–47. doi: 10.5664/jcsm.27032, PMID: 18198809 PMC2556918

[27] Stang A . Critical evaluation of the newcastle-ottawa scale for the assessment of the quality of nonrandomized studies in meta-analyses. Eur J Of Epidemiol. (2010) 25:603–5. doi: 10.1007/s10654-010-9491-z, PMID: 20652370

[28] Singh A Meshram H Srikanth M . American academy of sleep medicine guidelines, 2018. Int J Head Neck Surg. (2014) 10:102–3. doi: 10.5005/jp-journals-10001-1379

[29] Sharma P Thakur S Rai DK Karmakar S . Connecting the dots: analysing the relationship between ahi and odi in obstructive sleep apnea. Sleep Sci Pract. (2024) 8:9. doi: 10.1186/s41606-024-00102-x

[30] Shaw JE Punjabi NM Wilding JP Alberti KGMM Zimmet PZ . Sleep-disordered breathing and type 2 diabetes: A report from the international diabetes federation taskforce on epidemiology and prevention. Diabetes Res Clin Pract. (2008) 81:2–12. doi: 10.1016/j.diabres.2008.04.025, PMID: 18544448

[31] Andrassy KM . Comments on 'Kdigo 2012 clinical practice guideline for the evaluation and management of chronic kidney disease'. Kidney Int. (2013) 84:622–3. doi: 10.1038/ki.2013.243, PMID: 23989362

[32] Dong M Guo F Zhou T Wei Q . Association of diabetic nephropathy with the severity of obstructive sleep apnea-hypopnea syndrome in patients with type 2 diabetes mellitus. Endoc J. (2020) 67:515–22. doi: 10.1507/endocrj.EJ19-0324, PMID: 32023571

[33] Zhang P Zhang R Zhao F Heeley E Chai-Coetzer CL Liu J . The prevalence and characteristics of obstructive sleep apnea in hospitalized patients with type 2 diabetes in China. J sleep Res. (2016) 25:39–46. doi: 10.1111/jsr.12334, PMID: 26268508

[34] Yu W Wang X Ni Y Huai D Hao H Li Q . Association of osahs hypoxia indicators with early renal injury and serum fibroblast growth factor 21 in obese type 2 diabetic patients. Diabetes Ther: Res Treat Educ Diabetes Rel Disord. (2019) 10:1357–68. doi: 10.1007/s13300-019-0639-x, PMID: 31172456 PMC6612341

[35] Xue P Covassin N Ran X Zhou J Zhang X Yan D . Association of parameters of nocturnal hypoxemia with diabetic microvascular complications: A cross-sectional study. Diabetes Res Clin Pract. (2020) 170:108484. doi: 10.1016/j.diabres.2020.108484, PMID: 33031843

[36] Zhang R Guo X Guo L Lu J Zhou X Ji L . Prevalence and associated factors of obstructive sleep apnea in hospitalized patients with type 2 diabetes in beijing, China 2. J Diabetes. (2015) 7:16–23. doi: 10.1111/1753-0407.12180, PMID: 24910109

[37] Zhang R Zhang P Zhao F Han X Ji L . Association of diabetic microvascular complications and parameters of obstructive sleep apnea in patients with type 2 diabetes. Diabetes Technol Ther. (2016) 18:415–20. doi: 10.1089/dia.2015.0433, PMID: 27031372

[38] Stadler S Zimmermann T Franke F Rheinberger M Heid IM Böger CA . Association of sleep-disordered breathing with diabetes-associated kidney disease. Ann Med. (2017) 49:487–95. doi: 10.1080/07853890.2017.1306100, PMID: 28281834

[39] Banghoej AM Nerild HH Kristensen PL Pedersen-Bjergaard U Fleischer J Jensen AEK . Obstructive sleep apnoea is frequent in patients with type 1 diabetes. J Diabetes its Compl. (2017) 31:156–61. doi: 10.1016/j.jdiacomp.2016.10.006, PMID: 28029582

[40] Meyer L Massuyeau M Canel C Bahougne T Assemi P Perrin AE . Association of sleep apnoea syndrome and autonomic neuropathy in type 1 diabetes. Diabetes Metab. (2019) 45:206–9. doi: 10.1016/j.diabet.2017.10.011, PMID: 29169926

[41] Tahrani AA Ali A Raymond NT Begum S Dubb K Mughal S . Obstructive sleep apnea and diabetic neuropathy: A novel association in patients with type 2 diabetes. Am J Respir Crit Care Med. (2012) 186:434–41. doi: 10.1164/rccm.201112-2135OC, PMID: 22723291 PMC3443800

[42] Storgaard H Mortensen B Almdal T Laub M Tarnow LJDM . At least one in three people with type 2 diabetes mellitus referred to a diabetes centre has symptomatic obstructive sleep apnoea. Diabetic Medicine. (2014) 31:1460–7. doi: 10.1111/dme.12477, PMID: 24766227

[43] Tahrani AA Ali A Raymond NT Begum S Dubb K Altaf Q . Obstructive sleep apnea and diabetic nephropathy: A cohort study. Diabetes Care. (2013) 36:3718–25. doi: 10.2337/dc13-0450, PMID: 24062320 PMC3816897

[44] Nicholl DD Ahmed SB Loewen AH Hemmelgarn BR Sola DY Beecroft JM . Declining kidney function increases the prevalence of sleep apnea and nocturnal hypoxia. Chest. (2012) 141:1422–30. doi: 10.1378/chest.11-1809, PMID: 22222188

[45] Charlton A Garzarella J Jandeleit-Dahm KA Jha JC . Oxidative stress and inflammation in renal and cardiovascular complications of diabetes. Biology. (2020) 10:18. doi: 10.3390/biology10010018, PMID: 33396868 PMC7830433

[46] Arnardottir ES Mackiewicz M Gislason T Teff KL Pack AI . Molecular signatures of obstructive sleep apnea in adults: A review and perspective. Sleep. (2009) 32:447–70. doi: 10.1093/sleep/32.4.447, PMID: 19413140 PMC2663860

[47] Pérez-Torres I Soto ME Manzano-Pech L Díaz-Diaz E Soria-Castro E Rubio-Ruíz ME . Oxidative stress in plasma from patients with marfan syndrome is modulated by deodorized garlic preliminary findings. Oxid Med And Cell Longevity. (2022) 2022:5492127. doi: 10.1155/2022/5492127, PMID: 35082968 PMC8786463

[48] JhaJay C ChowBryna S CooperMark E . Diabetes and kidney disease: role of oxidative stress. Antioxidants Redox Signaling. (2016) 25:657–684. doi: 10.1089/ars.2016.6664, PMID: 26906673 PMC5069735

[49] Chen PS Chiu WT Hsu PL Lin SC Peng IC Wang CY . Pathophysiological implications of hypoxia in human diseases. J Biomed Sci. (2020) 27:63. doi: 10.1186/s12929-020-00658-7, PMID: 32389123 PMC7212687

[50] Kang HH Kim IK Lee H Joo H JU L Lee J . Chronic intermittent hypoxia induces liver fibrosis in mice with diet-induced obesity via tlr4/myd88/mapk/nf-kb signaling pathways. Biochem Biophys Res Commun. (2017) 490:349–55. doi: 10.1016/j.bbrc.2017.06.047, PMID: 28623125

[51] Gulotta G Iannella G Vicini C Polimeni A Greco A de Vincentiis M . Risk factors for obstructive sleep apnea syndrome in children: state of the art. Int J Environ Res Public Health. (2019) 16:3235. doi: 10.3390/ijerph16183235, PMID: 31487798 PMC6765844

[52] Kheirandish-Gozal L Gozal D . Obstructive sleep apnea and inflammation: proof of concept based on two illustrative cytokines. Int J Mol Sci. (2019) 20:459. doi: 10.3390/ijms20030459, PMID: 30678164 PMC6387387

[53] Rayego-Mateos S Morgado-Pascual JL Opazo-Ríos L Guerrero-Hue M García-Caballero C Vázquez-Carballo C . Pathogenic pathways and therapeutic approaches targeting inflammation in diabetic nephropathy. Int J Of Mol Sci. (2020) 21:3798. doi: 10.3390/ijms21113798, PMID: 32471207 PMC7312633

[54] Zhang LL Jiang FM Xie YY Mo Y Zhang X Liu CT . Diabetic endothelial microangiopathy and pulmonary dysfunction. Front Endocrinol. (2023) 14:1073878. doi: 10.3389/fendo.2023.1073878, PMID: 37025413 PMC10071002

[55] Lavie L . Oxidative stress inflammation and endothelial dysfunction in obstructive sleep apnea. Front Biosci-Elite. (2012) 4:1391–403. doi: 10.2741/e469 22201964

[56] Lévy P Kohler M McNicholas WT Barbé F McEvoy RD Somers VK . Obstructive sleep apnoea syndrome. Nat Rev Dis Primers. (2015) 1:1–21. doi: 10.1038/nrdp.2015.15, PMID: 27188535

[57] Ricardo AC Knutson K Chen J Appel LJ Bazzano L Carmona-Powell E . The association of sleep duration and quality with ckd progression. J Of Am Soc Of Nephrol. (2017) 28:3708–15. doi: 10.1681/ASN.2016121288, PMID: 28912373 PMC5698066

[58] Hamilton GS Naughton MT . Impact of obstructive sleep apnoea on diabetes and cardiovascular disease. Med J Aust. (2013) 199:S27–30. doi: 10.5694/mja13.10579, PMID: 24138362

[59] Ray R Juranek JK Rai V . Rage axis in neuroinflammation, neurodegeneration and its emerging role in the pathogenesis of amyotrophic lateral sclerosis. Neurosci And Biobehav Rev. (2016) 62:48–55. doi: 10.1016/j.neubiorev.2015.12.006, PMID: 26724598

[60] Mendoza JM Isakova T Cai X Bayes LY Faul C Scialla JJ . Inflammation and elevated levels of fibroblast growth factor 23 are independent risk factors for death in chronic kidney disease. Kidney Int. (2017) 91:711–9. doi: 10.1016/j.kint.2016.10.021, PMID: 28017325 PMC5313324

[61] Edmonston D Wojdyla D Mehta R Cai X Lora C Cohen D . Single measurements of carboxy-terminal fibroblast growth factor 23 and clinical risk prediction of adverse outcomes in ckd. Am J Of Kidney Dis. (2019) 74:771–81. doi: 10.1053/j.ajkd.2019.05.026, PMID: 31445926 PMC6875624

[62] Lin CH Lurie RC Lyons OD . Sleep apnea and chronic kidney disease a state-of-the-art review. Chest. (2020) 157:673–85. doi: 10.1016/j.chest.2019.09.004, PMID: 31542452

[63] Elias RM Chan CT Paul N Motwani SS Kasai T Gabriel JM . Relationship of pharyngeal water content and jugular volume with severity of obstructive sleep apnea in renal failure. Nephrol Dialysis Transplant. (2013) 28:937–44. doi: 10.1093/ndt/gfs473, PMID: 23136217

[64] Voulgaris A Bonsignore MR Schiza S Marrone O Steiropoulos P . Is kidney a new organ target in patients with obstructive sleep apnea? Research priorities in a rapidly evolving field. Sleep Med. (2021) 86:56–67. doi: 10.1016/j.sleep.2021.08.009, PMID: 34474225

[65] Duffin J . Role of acid-base balance in the chemoreflex control of breathing. J Of Appl Physiol. (2005) 99:2255–65. doi: 10.1152/japplphysiol.00640.2005, PMID: 16109829

[66] Beecroft J Duffin J Pierratos A Chan CT McFarlane P Hanly PJ . Enhanced chemo-responsiveness in patients with sleep apnoea and end-stage renal disease. Eur Respir J. (2006) 28:151–8. doi: 10.1183/09031936.06.00075405, PMID: 16510459

[67] Rosen CL Debaun MR Strunk RC Redline S Seicean S Craven DI . Obstructive sleep apnea and sickle cell anemia. Pediatrics. (2014) 134:273–81. doi: 10.1542/peds.2013-4223, PMID: 25022740 PMC4187233

[68] Turek NF Ricardo AC Lash JP . Sleep disturbances as nontraditional risk factors for development and progression of ckd: review of the evidence. Am J Of Kidney Dis. (2012) 60:823–33. doi: 10.1053/j.ajkd.2012.04.027, PMID: 22727724 PMC3461247

[69] Leong WB Jadhakhan F Taheri S Thomas GN Adab P . The association between obstructive sleep apnea on diabetic kidney disease: A systematic review and meta-analysis. Sleep. (2016) 39:301–8. doi: 10.5665/sleep.5432%JSleep, PMID: 26414891 PMC4712397

