# Supplementary material for: The bidirectional association between obstructive sleep apnea and diabetic kidney disease: systematic review and meta-analysis
Source: Front Endocrinol (Lausanne). 2025 Nov 24;16:1715997. doi: 10.3389/fendo.2025.1715997 (PMC12682665; doi:10.3389/fendo.2025.1715997)

Supplementary Materials

	Content	Page
**Table S1.**	PRISMA 2020 checklist	**2-5**
**Table S2.**	Representatives search strings for PubMed	**6**
**Table S3-S10.**	Results of sensitivity analyses	**7-9**
**Figure S1**	Forest plot of the association between severe OSA and DKD after excluding one study	**9**
**Figure S2**	Forest plot of the association between the severity of proteinuria in DKD and AHI after excluding one study	**10**
**Figure S3**	Forest Plot of the association between proteinuria severity and L-SaO_2 in DKD	**10**
**Figure S4**	GRADE Assessment of Evidence Quality for OSA and DKD Associations	**11**
**Figure S5-S12**	Prediction Interval Plot (Including Fixed Effects Model and Random Effects Model)	**12-15**

Table S1. PRISMA 2020 checklist

Section and Topic	Item #	Checklist item	Location where item is reported
**TITLE**			
Title	1	Identify the report as a systematic review.	P1
**ABSTRACT**			
Abstract	2	See the PRISMA 2020 for Abstracts checklist.	P1-2
**INTRODUCTION**			
Rationale	3	Describe the rationale for the review in the context of existing knowledge.	P2-3
Objectives	4	Provide an explicit statement of the objective(s) or question(s) the review addresses.	P3
**METHODS**			
Eligibility criteria	5	Specify the inclusion and exclusion criteria for the review and how studies were grouped for the syntheses.	P3
Information sources	6	Specify all databases, registers, websites, organisations, reference lists and other sources searched or consulted to identify studies. Specify the date when each source was last searched or consulted.	P3
Search strategy	7	Present the full search strategies for all databases, registers and websites, including any filters and limits used.	P4
Selection process	8	Specify the methods used to decide whether a study met the inclusion criteria of the review, including how many reviewers screened each record and each report retrieved, whether they worked independently, and if applicable, details of automation tools used in the process.	P5
Data collection process	9	Specify the methods used to collect data from reports, including how many reviewers collected data from each report, whether they worked independently, any processes for obtaining or confirming data from study investigators, and if applicable, details of automation tools used in the process.	P5-6
Data items	10a	List and define all outcomes for which data were sought. Specify whether all results that were compatible with each outcome domain in each study were sought (e.g. for all measures, time points, analyses), and if not, the methods used to decide which results to collect.	P4-5

	10b	List and define all other variables for which data were sought (e.g. participant and intervention characteristics, funding sources). Describe any assumptions made about any missing or unclear information.	P4
Study risk of bias assessment	11	Specify the methods used to assess risk of bias in the included studies, including details of the tool(s) used, how many reviewers assessed each study and whether they worked independently, and if applicable, details of automation tools used in the process.	P6
Effect measures	12	Specify for each outcome the effect measure(s) (e.g. risk ratio, mean difference) used in the synthesis or presentation of results.	P5
Synthesis methods	13a	Describe the processes used to decide which studies were eligible for each synthesis (e.g. tabulating the study intervention characteristics and comparing against the planned groups for each synthesis (item #5)).	P5
	13b	Describe any methods required to prepare the data for presentation or synthesis, such as handling of missing summary statistics, or data conversions.	P4-5
	13c	Describe any methods used to tabulate or visually display results of individual studies and syntheses.	P5
	13d	Describe any methods used to synthesize results and provide a rationale for the choice(s). If meta-analysis was performed, describe the model(s), method(s) to identify the presence and extent of statistical heterogeneity, and software package(s) used.	P5
	13e	Describe any methods used to explore possible causes of heterogeneity among study results (e.g. subgroup analysis, meta-regression).	P5
	13f	Describe any sensitivity analyses conducted to assess robustness of the synthesized results.	P5
Reporting bias assessment	14	Describe any methods used to assess risk of bias due to missing results in a synthesis (arising from reporting biases).	P8
**Section and Topic**	**Item #**	**Checklist item**	**Location where item is reported**
Certainty assessment	15	Describe any methods used to assess certainty (or confidence) in the body of evidence for an outcome.	P8
**RESULTS**			
Study selection	16a	Describe the results of the search and selection process, from the number of records identified in the search to the number of studies included in the review, ideally using a flow diagram.	P5, figure 1

	16b	Cite studies that might appear to meet the inclusion criteria, but which were excluded, and explain why they were excluded.	P8, figure 1
Study characteristics	17	Cite each included study and present its characteristics.	P5-6, Table1
Risk of bias in studies	18	Present assessments of risk of bias for each included study.	P8, Figure 8
Results of individual studies	19	For all outcomes, present, for each study: (a) summary statistics for each group (where appropriate) and (b) an effect estimate and its precision (e.g. confidence/credible interval), ideally using structured tables or plots.	P6-8, Figure 2-7, Supplementary Figure 1-2
Results of syntheses	20a	For each synthesis, briefly summarise the characteristics and risk of bias among contributing studies.	P8
	20b	Present results of all statistical syntheses conducted. If meta-analysis was done, present for each the summary estimate and its precision (e.g. confidence/credible interval) and measures of statistical heterogeneity. If comparing groups, describe the direction of the effect.	P6-8,.
	20c	Present results of all investigations of possible causes of heterogeneity among study results.	P6-8
	20d	Present results of all sensitivity analyses conducted to assess the robustness of the synthesized results.	P8,Table S3-S10
Reporting biases	21	Present assessments of risk of bias due to missing results (arising from reporting biases) for each synthesis assessed.	P8
Certainty of evidence	22	Present assessments of certainty (or confidence) in the body of evidence for each outcome assessed.	P6-8
**DISCUSSION**			
Discussion	23a	Provide a general interpretation of the results in the context of other evidence.	P8-10
	23b	Discuss any limitations of the evidence included in the review.	P11-12
	23c	Discuss any limitations of the review processes used.	P12
	23d	Discuss implications of the results for practice, policy, and future research.	P11-12
**OTHER INFORMATION**			
Registration and	24a	Provide registration information for the review, including register name and registration number, or state that the review was not registered.	P3,CRD420251157002

protocol	24b	Indicate where the review protocol can be accessed, or state that a protocol was not prepared.	P3,CRD42025115 7002
	24c	Describe and explain any amendments to information provided at registration or in the protocol.	
Support	25	Describe sources of financial or non-financial support for the review, and the role of the funders or sponsors in the review.	
Competing interests	26	Declare any competing interests of review authors.	P12
Availability of data, code and other materials	27	Report which of the following are publicly available and where they can be found: template data collection forms; data extracted from included studies; data used for all analyses; analytic code; any other materials used in the review.	P12

From: Page MJ, McKenzie JE, Bossuyt PM, et al. The PRISMA 2020 statement: an updated guideline for reporting systematic reviews. *BMJ* 2021;372:n71. doi:10.1136/bmj.n71
For more information, visit: **www.prisma-statement.org**.

Table S2. Representatives search strings for PubMed (From inception to September 2025)	
**Databases**	**Search strings**
**PubMed**	#1"Diabetic Nephropathies"[Mesh]<br>#2(((diabetic kidney disease[Title/Abstract]) OR (Nephropathies, Diabetic[Title/Abstract])) OR (Kidney Diseases, Diabetic[Title/Abstract])) OR (Diabetic Nephropathy[Title/Abstract]) Sort by: Publication Date<br>#3 #1OR#2<br>#4 ((((((((obstructive sleep apnea[Title/Abstract]) OR (obstructive sleep apnoea[Title/Abstract])) OR (sleep apnea syndrome[Title/Abstract])) OR (sleep apnoea syndrome[Title/Abstract])) OR (sleep-disordered breathing[Title/Abstract])) OR (OSA[Title/Abstract])) OR (SAS[Title/Abstract])) OR (SDB[Title/Abstract])) OR (OSAS[Title/Abstract]))

Table S3. Sensitivity Analysis Results on the Correlation Between OSA and DKD Incidence

Studies omitted	OR (95% CI)	$P_{association}$	Heterogeneity
Banghoej 2017	1.92 [1.58, 2.34]	P<0.00001	15%
Dong 2020	1.87 [1.55, 2.27]	P<0.00001	4%
Furukawa 2013	1.85 [1.52, 2.24]	P<0.00001	0%
Meyer 2019	1.92 [1.59, 2.32]	P<0.00001	15%
Storgaard 2014	1.98 [1.63, 2.41]	P<0.00001	5%
Tahrani 2012	1.88 [1.54, 2.28]	P<0.00001	9%
Tahrani 2013	1.82 [1.50, 2.22]	P<0.00001	0%
Xue 2020	1.93 [1.60, 2.33]	P<0.00001	13%
Zhang 2015	1.92 [1.59, 2.31]	P<0.00001	14%
Zhang 2016	2.29 [1.78, 2.93]	P<0.00001	0%

Table S4. Sensitivity Analysis Results of the Association Between Mild-to-Moderate OSA and DKD

Studies omitted	OR (95% CI)	$P_{association}$	Heterogeneity
Dong 2020	1.50 [1.20, 1.89]	P = 0.0005	0%
Stadler 2017	1.87 [1.55, 2.27]	P = 0.003	0%
Xue 2020	1.52 [1.22, 1.91]	P = 0.0002	0%
Zhang 2016	1.52 [1.09, 2.13]	P = 0.01	0%

Table S5. Sensitivity Analysis Results of the Association Between Severe OSA and DKD

Studies omitted	OR (95% CI)	$P_{association}$	Heterogeneity
Dong 2020	2.03 [1.19, 3.44]	P = 0.009	44%
Stadler 2017	2.14 [1.00, 4.55]	P = 0.05	54%
Xue 2020	2.51 [1.39, 4.54]	P = 0.002	65%
Zhang 2016	3.09 [2.00, 4.79]	P<0.00001	0%

Table S6. Sensitivity Analysis Results for the Association Between OSA and eGFR

Studies omitted	MD (95% CI)	$P_{association}$	Heterogeneity
Tahrani 2012	-7.36 [-12.91, -1.82]	P = 0.009	54%
Tahrani 2013	-7.39 [-12.81, -1.97]	P = 0.008	53%
Yu 2019	-10.60[-15.54, -5.66]	P<0.001	0%

Table S7. Sensitivity Analysis Results on the Correlation Between DKD and OSA Incidence

Studies omitted	OR (95% CI)	$P_{association}$	Heterogeneity
Leong 2014	1.77 [0.68, 4.67]	P = 0.24	0%
Xue 2020	1.63 [0.66, 4.04]	P = 0.29	0%
Yu 2019	1.24 [0.44, 3.53]	P = 0.69	0%

Table S8. Sensitivity Analysis Results of the Association Between Proteinuria Severity and AHI in DKD

Studies omitted	MD (95% CI)	$P_{association}$	Heterogeneity
Leong 2014	6.75 (1.80, 11.70)	P = 0.008	87%
Xue 2020	7.88 (2.24, 13.52)	P = 0.006	86%
Yu 2019	6.07 (0.94, 11.21)	P = 0.02	85%
Dong 2020 (all subgroups)	1.93 (-0.65, 4.52)	P = 0.14	43%
Zhang R 2016 (all subgroups)	9.79 (1.66, 17.93)	P = 0.02	81%

Table S9. Sensitivity Analysis Results of the Association Between Proteinuria Severity and L-SaO_2 in DKD

Studies omitted	MD (95% CI)	$P_{association}$	Heterogeneity
Leong 2014	-0.39 (-1.22, 0.43)	P = 0.35	54%
Xue 2020	-0.48 (-1.35, 0.39)	P = 0.28	57%
Yu 2019	-0.32 (-1.15, 0.50)	P = 0.44	43%
Dong 2020 (all subgroups)	-0.21 (-1.05, 0.62)	P = 0.62	13%
Zhang R 2016 (all subgroups)	-1.95 (-3.53, -0.38)	P = 0.01	37%

Table S10. Sensitivity Analysis Results of the Association Between Proteinuria Severity and M-SaO_2 in DKD

Studies omitted	MD (95% CI)	$P_{association}$	Heterogeneity
Leong 2014	-0.57 (-0.80, -0.34)	P<0.00001	33%
Xue 2020	-0.62 (-0.86, -0.39)	P<0.00001	27%
Yu 2019	-0.49 (-0.74, -0.23)	P = 0.0002	0%
Dong 2020 (all subgroups)	-0.56 (-0.79, -0.33)	P<0.00001	27%
Zhang R 2016 (all subgroups)	-0.88 (-1.27, -0.48)	P<0.00001	14%

Figure S1: Forest plot of the association between severe OSA and DKD after excluding one study

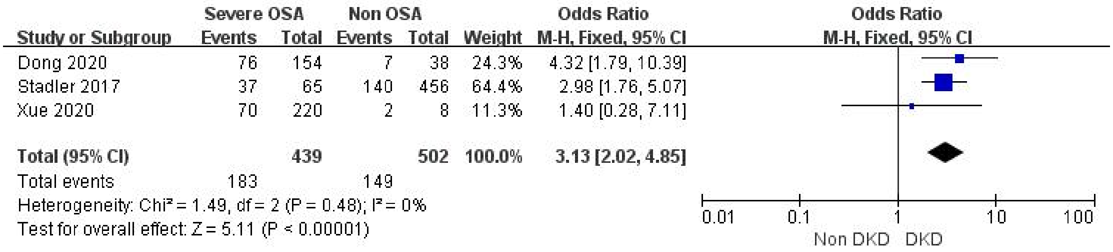

Figure S2: Forest plot of the association between the severity of proteinuria in DKD and AHI after excluding one study

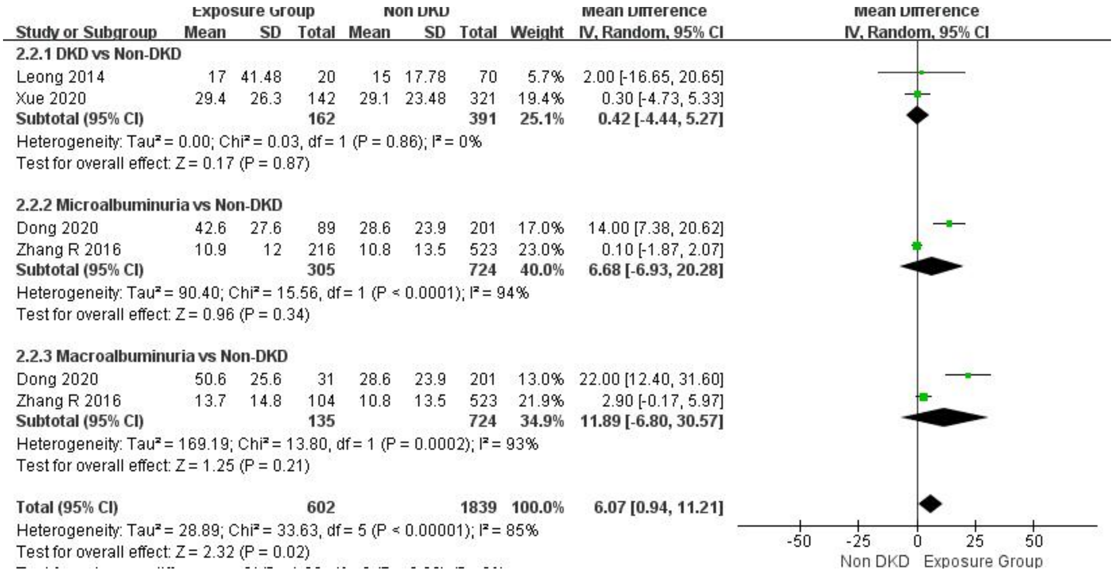

Figure S3: Forest Plot of the association between proteinuria severity and L-SaO_2 in DKD

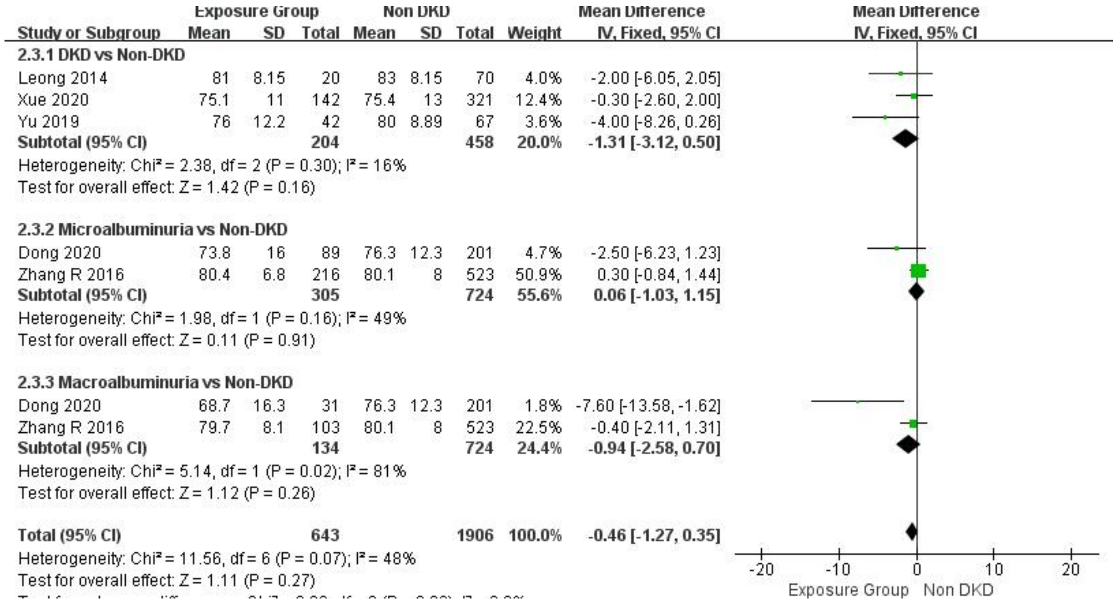

Figure S4: GRADE Assessment of Evidence Quality for OSA and DKD Associations

Author(s):
Date: 2025-10-25
Question: Should OSA be used for DKD?
Settings:
Bibliography: . OSA for DKD. Cochrane Database of Systematic Reviews [Year], Issue [Issue].

Quality assessment							No of patients		Effect		Quality	Importance
No of studies	Design	Risk of bias	Inconsistency	Indirectness	Imprecision	Other considerations	OSA	Control	Relative (95% CI)	Absolute		
Correlation between OSA and the Incidence of DKD												
10	randomised trials	no serious risk of bias	no serious inconsistency	no serious indirectness	no serious imprecision[1]	none	727/2276 (31.9%)	251/1241 (20.2%)	OR 1.92 (1.59 to 2.32)	125 more per 1000 (from 85 more to 168 more)	⊕⊕⊕⊕ HIGH	CRITICAL
								23.9%		137 more per 1000 (from 94 more to 183 more)		
Association between mild to severe OSA and DKD												
4	randomised trials	no serious risk of bias	no serious inconsistency[1]	no serious indirectness	no serious imprecision	none	219/523 (41.9%)	261/841 (31%)	OR 2.29 (1.66 to 3.16)	197 more per 1000 (from 117 more to 277 more)	⊕⊕⊕⊕ HIGH	CRITICAL
								27.9%		191 more per 1000 (from 112 more to 271 more)		
Association between mild to moderate OSA and DKD												
4	randomised trials	no serious risk of bias	no serious inconsistency	no serious indirectness	no serious imprecision	none	359/965 (37.2%)	261/841 (31%)	OR 1.52 (1.22 to 1.9)	96 more per 1000 (from 44 more to 151 more)	⊕⊕⊕⊕ HIGH	CRITICAL
								27.9%		91 more per 1000 (from 42 more to 145 more)		
Association between OSA and eGFR (Better indicated by lower values)												
3	randomised trials	no serious risk of bias	no serious inconsistency	no serious indirectness	serious[1]	none	388	179	-	MD 8.61 lower (12.92 to 4.3 lower)	⊕⊕⊕O MODERATE	CRITICAL

[1] 1No explanation was provided

Author(s):
Date: 2025-10-25
Question: Should DKD be used for OSA?
Settings:
Bibliography: . OSA for DKD. Cochrane Database of Systematic Reviews [Year], Issue [Issue].

Quality assessment							No of patients		Effect		Quality	Importance
No of studies	Design	Risk of bias	Inconsistency	Indirectness	Imprecision	Other considerations	DKD	Control	Relative (95% CI)	Absolute		
Correlation between DKD and the Incidence of OSA												
3	randomised trials	no serious risk of bias	no serious inconsistency	no serious indirectness	serious[1]	none	195/204 (95.6%)	428/458 (93.4%)	OR 1.56 (0.71 to 3.43)	23 more per 1000 (from 24 fewer to 45 more)	⊕⊕⊕O MODERATE	CRITICAL
								82.9%		54 more per 1000 (from 54 fewer to 114 more)		
Association between severity of proteinuria in DKD and AHI (Better indicated by lower values)												
5	randomised trials	no serious risk of bias	very serious[2]	no serious indirectness	no serious imprecision	none	644	1906	-	MD 6.48 higher (1.74 to 11.22 higher)	⊕⊕OO LOW	IMPORTANT
DKD与L-SaO2 (Better indicated by lower values)												
5	randomised trials	no serious risk of bias	serious[2]	no serious indirectness	no serious imprecision[2]	none	643	1906	-	MD 0.46 lower (1.27 lower to 0.35 higher)	⊕⊕⊕O MODERATE	IMPORTANT
DKD与M-SaO2 (Better indicated by lower values)												
5	randomised trials	no serious risk of bias	serious[2]	no serious indirectness	no serious imprecision[2]	none	643	1906	-	MD 0.59 lower (0.82 to 0.36 lower)	⊕⊕⊕O MODERATE	IMPORTANT

[1] No explanation was provided
[2] 11

Figure S5: Prediction Interval Plot for Correlation between OSA and the Incidence of DKD

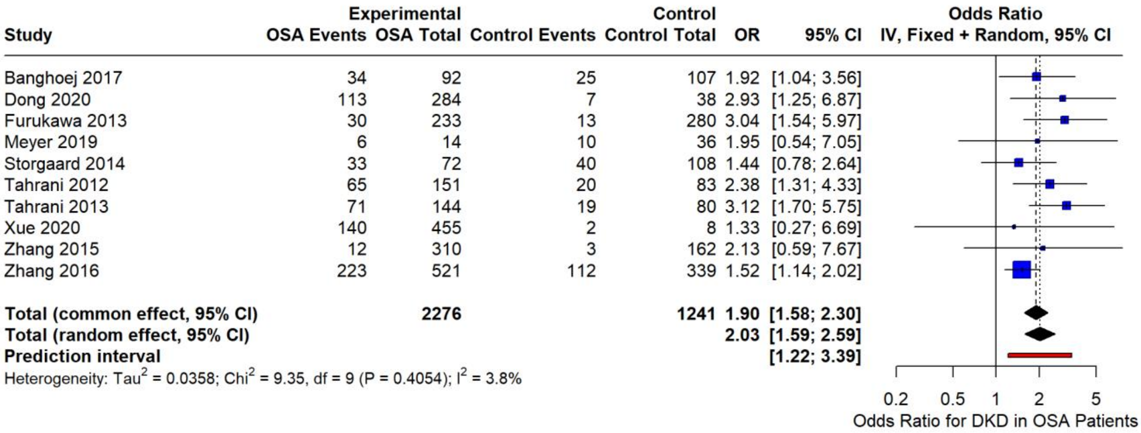

Figure S6: Prediction Interval Plot for the Association Between Mild-to-Moderate OSA and DKD

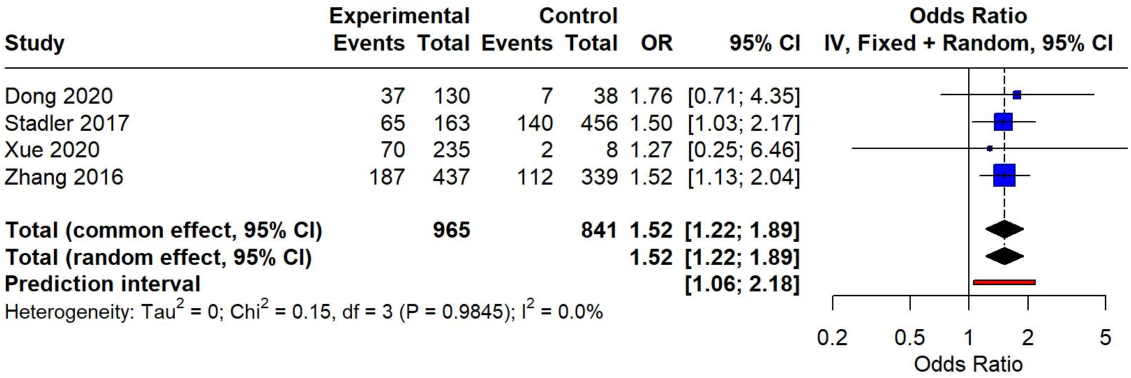

Figure S7: Prediction Interval Plot for the Association Between Severe OSA and DKD

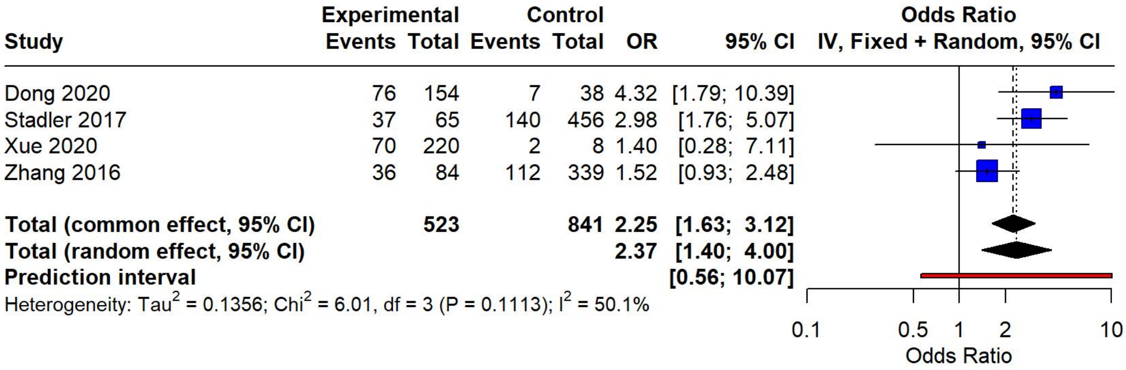

Figure S8: Prediction Interval Plot for the Association Between OSA and eGFR

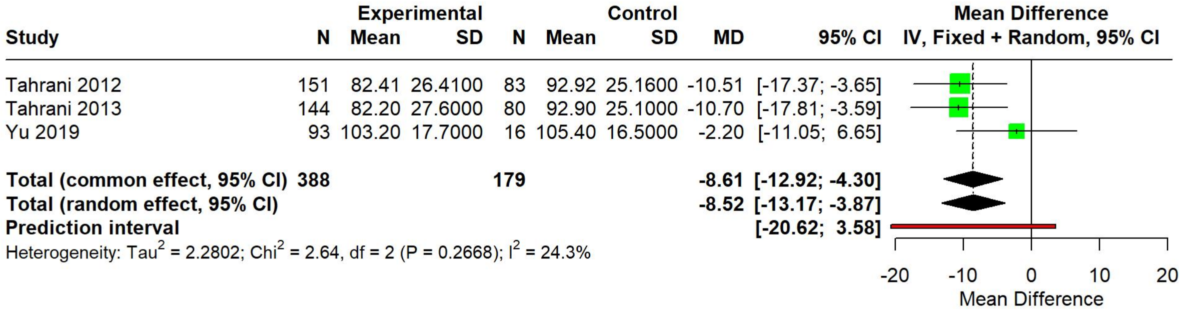

Figure S9: Prediction Interval Plot for the Correlation Between DKD and the Incidence of OSA

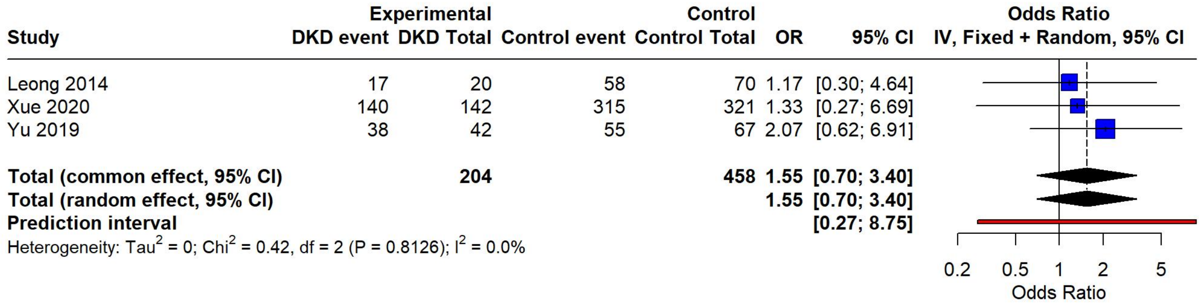

Figure S10: Prediction Interval Plot for the Association Between Severity of Proteinuria in DKD and AHI

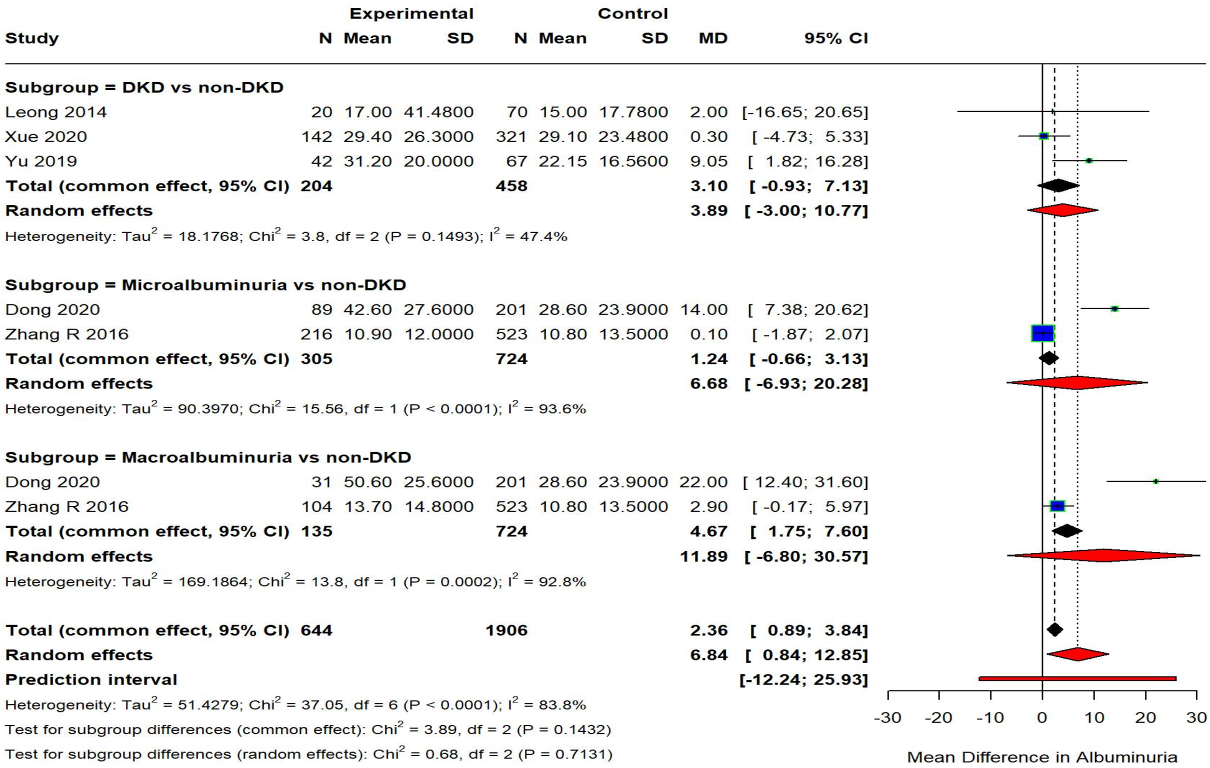

Figure S11: Prediction Interval Plot for the Association Between Proteinuria Severity in DKD and L-SaO_2

	Experimental			Control				
**Study**	**N**	**Mean**	**SD**	**N**	**Mean**	**SD**	**MD**	**95% CI**
**Subgroup = DKD vs non-DKD**								
Leong 2014	20	81.00	8.1500	70	83.00	8.1500	-2.00	[-6.05; 2.05]
Xue 2020	142	75.10	11.0000	321	75.40	13.0000	-0.30	[-2.60; 2.00]
Yu 2019	42	76.00	12.2000	67	80.00	8.8900	-4.00	[-8.26; 0.26]
**Total (common effect, 95% CI)**	**204**			**458**			**-1.31**	**[-3.12; 0.50]**
**Random effects**							**-1.52**	**[-3.70; 0.66]**
Heterogeneity: Tau^2 = 0.9140; Chi^2 = 2.38, df = 2 (P = 0.3037); I^2 = 16.1%								
**Subgroup = Microalbuminuria vs non-DKD**								
Dong 2020	89	73.80	16.0000	201	76.30	12.3000	-2.50	[-6.23; 1.23]
Zhang R 2016	216	80.40	6.8000	523	80.10	8.0000	0.30	[-0.84; 1.44]
**Total (common effect, 95% CI)**	**305**			**724**			**0.06**	**[-1.03; 1.15]**
**Random effects**							**-0.51**	**[-3.00; 1.98]**
Heterogeneity: Tau^2 = 1.9372; Chi^2 = 1.98, df = 1 (P = 0.1597); I^2 = 49.4%								
**Subgroup = Macroalbuminuria vs non-DKD**								
Dong 2020	31	68.70	16.3000	201	76.30	12.3000	-7.60	[-13.58; -1.62]
Zhang R 2016	103	79.70	8.1000	523	80.10	8.0000	-0.40	[-2.11; 1.31]
**Total (common effect, 95% CI)**	**134**			**724**			**-0.94**	**[-2.58; 0.70]**
**Random effects**							**-3.41**	**[-10.36; 3.55]**
Heterogeneity: Tau^2 = 20.8787; Chi^2 = 5.14, df = 1 (P = 0.0234); I^2 = 80.6%								
**Total (common effect, 95% CI)**	**643**			**1906**			**-0.46**	**[-1.27; 0.35]**
**Random effects**							**-1.10**	**[-2.42; 0.23]**
**Prediction interval**								**[-4.25; 2.06]**

Heterogeneity: Tau^2 = 1.2105; Chi^2 = 11.56, df = 6 (P = 0.0725); I^2 = 48.1%

Test for subgroup differences (common effect): Chi^2 = 2.06, df = 2 (P = 0.3568)

Test for subgroup differences (random effects): Chi^2 = 0.76, df = 2 (P = 0.6833)

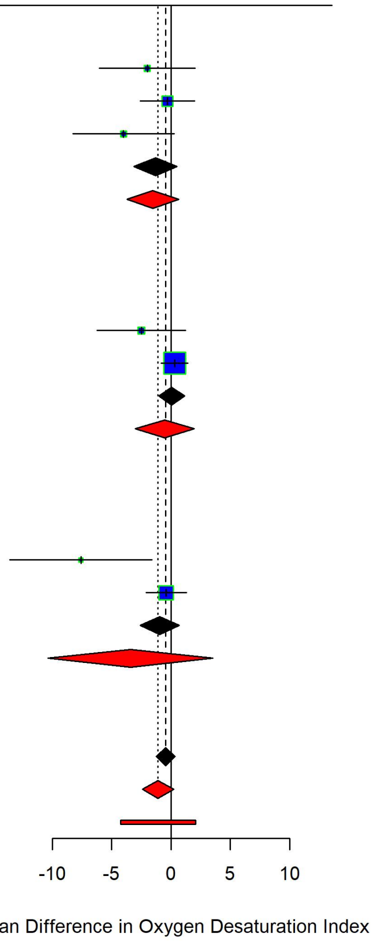

Figure S12: Prediction Interval Plot for the Association Between Proteinuria Severity in DKD and M-SaO_2

Study	Experimental N	Experimental Mean	Experimental SD	Control N	Control Mean	Control SD	MD	95% CI
**Subgroup = DKD vs non-DKD**								
Leong 2014	20	92.00	2.2200	70	93.00	2.2200	-1.00	[-2.10; 0.10]
Xue 2020	142	92.60	4.6000	321	92.70	4.1000	-0.10	[-0.98; 0.78]
Yu 2019	42	95.00	1.2300	67	96.00	1.4800	-1.00	[-1.51; -0.49]
**Total (common effect, 95% CI)**	**204**			**458**			**-0.80**	**[-1.21; -0.39]**
**Random effects**							**-0.74**	**[-1.32; -0.17]**
Heterogeneity: Tau^2 = 0.1021; Chi^2 = 3.14, df = 2 (P = 0.2079); I^2 = 36.3%								
**Subgroup = Microalbuminuria vs non-DKD**								
Dong 2020	89	91.40	10.1000	201	93.00	5.0000	-1.60	[-3.81; 0.61]
Zhang R 2016	216	93.00	2.0000	523	93.50	2.2000	-0.50	[-0.83; -0.17]
**Total (common effect, 95% CI)**	**305**			**724**			**-0.52**	**[-0.85; -0.20]**
**Random effects**							**-0.52**	**[-0.85; -0.20]**
Heterogeneity: Tau^2 = 0; Chi^2 = 0.93, df = 1 (P = 0.3344); I^2 = 0%								
**Subgroup = Macroalbuminuria vs non-DKD**								
Dong 2020	31	91.20	4.8000	201	93.00	5.0000	-1.80	[-3.63; 0.03]
Zhang R 2016	103	93.20	2.6000	523	93.50	2.2000	-0.30	[-0.84; 0.24]
**Total (common effect, 95% CI)**	**134**			**724**			**-0.42**	**[-0.93; 0.10]**
**Random effects**							**-0.79**	**[-2.16; 0.59]**
Heterogeneity: Tau^2 = 0.6538; Chi^2 = 2.39, df = 1 (P = 0.1223); I^2 = 58.1%								
**Total (common effect, 95% CI)**	**643**			**1906**			**-0.59**	**[-0.82; -0.36]**
**Random effects**							**-0.62**	**[-0.92; -0.32]**
**Prediction interval**								**[-1.22; -0.02]**
Heterogeneity: Tau^2 = 0.0369; Chi^2 = 8.07, df = 6 (P = 0.2327); I^2 = 25.7%								
Test for subgroup differences (common effect): Chi^2 = 1.61, df = 2 (P = 0.4463)								
Test for subgroup differences (random effects): Chi^2 = 0.51, df = 2 (P = 0.7754)								

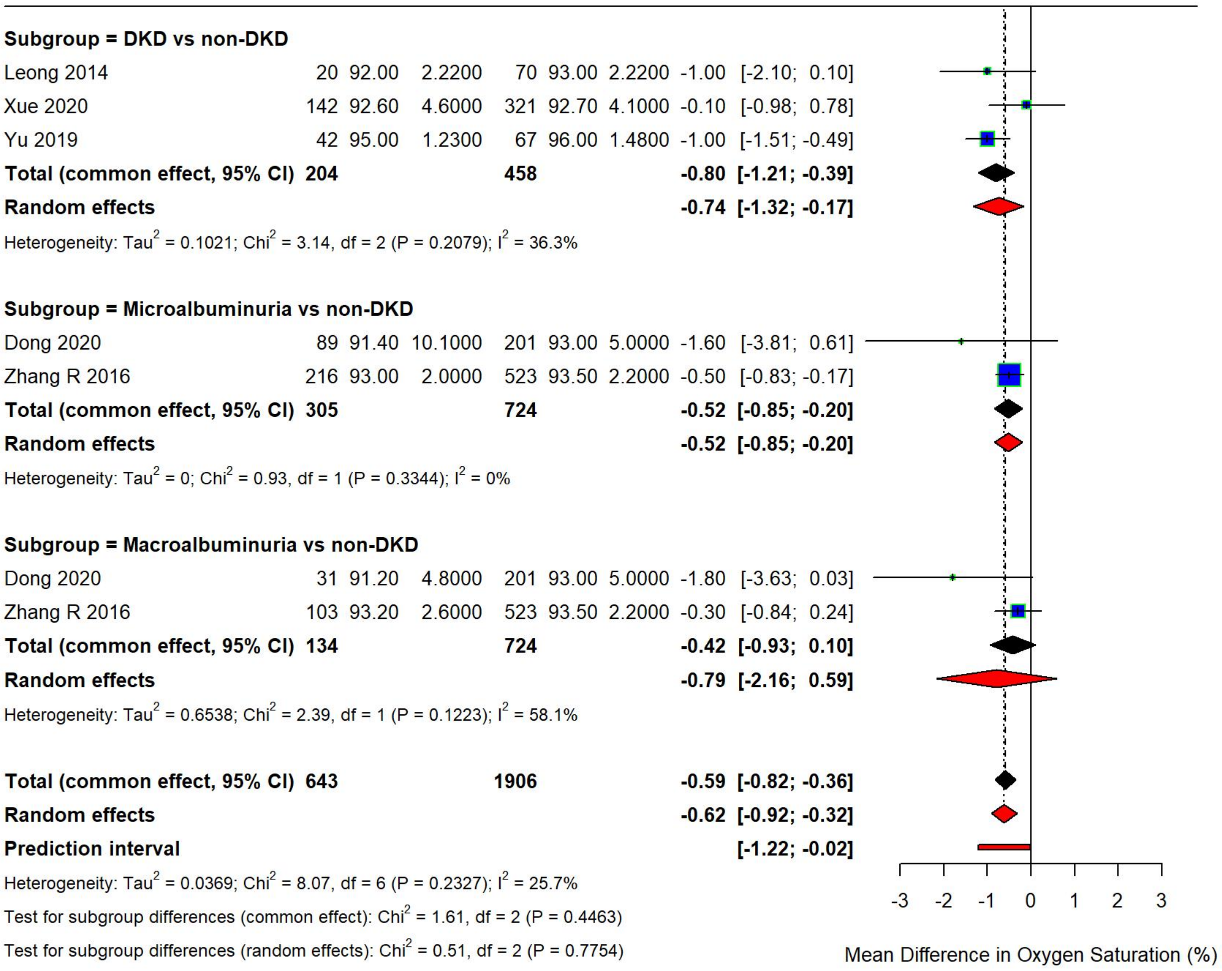

Supplement: Supplementary file 1 [file DataSheet1.pdf]
